# Comparative genomics and phenotypic studies to determine site-specificity of *Escherichia coli* in the lower gastrointestinal tract of humans

**DOI:** 10.1080/19490976.2023.2223332

**Published:** 2023-06-20

**Authors:** Rasel Barua, Paul Pavli, David M Gordon, Claire L O’Brien

**Affiliations:** a College of Health and Medicine, Australian National University, ACT, Canberra, Australia; b Research School of Biology, Australian National University, Canberra, Australia; c IBD Research Laboratory, Canberra Hospital, ACT, Canberra, Australia; d Gastroenterology and Hepatology Unit, Canberra Hospital, ACT, Canberra, Australia; e Centre for Research in Therapeutic Solutions, Faculty of Science and Technology, University of Canberra, ACT, Canberra, Australia

**Keywords:** *E. coli*, site-specificity, human gut, terminal ileum, rectum, adaptation

## Abstract

*Escherichia coli* (*E. coli*) is an important commensal in the human gut; however, it is unknown whether strains show site-specificity in the lower gut. To investigate this, we assessed genotypic and phenotypic differences in 37 clone pairs (two strains with very similar multiple locus variable-number-tandem-repeat analysis [MLVA] profiles) of *E. coli* isolated from mucosal biopsies of two different gut locations (terminal ileum and rectum). The clone pairs varied at the genomic level; single nucleotide polymorphisms (SNPs) were common, multiple nucleotide polymorphisms (MNPs) were observed but less common, and few indels (insertions and deletions) were detected. The variation was higher in clone pairs that are associated with non-human-associated sequence types (ST) compared to human-associated STs, such as ST95, ST131, and ST73. No gene(s) with non-synonymous mutations were found to be commonly associated with either the terminal ileum or the rectal strains. At the phenotypic level, we identified the metabolic signatures for some STs. Rectum strains of some STs showed consistently higher metabolic activity with particular carbon sources. Clone pairs belonging to specific STs showed distinct growth patterns under different pH conditions. Overall, this study showed that *E*. *coli* may exhibit genomic and phenotypic variability at different locations in the gut. Although genomics did not reveal significant information suggesting the site-specificity of strains, some phenotypic studies have suggested that strains may display site-specificity in the lower gut. These results provide insights into the nature and adaptation of *E. coli* in the lower gut of humans. To the best of our knowledge, no study has investigated or demonstrated the site-specificity of commensal *E. coli* in the human gut.

## Introduction

The site-specificity of commensal *Escherichia coli* (*E. coli*) in the human gut is poorly understood. Although several studies have examined the distribution of *E. coli* in the human gut, only one study has analyzed and compared *E. coli* from multiple locations in the lower gut.^1–5^ This study found that, in some individuals, *E. coli* strains were detected in only one gut location, for example, in the ileum, but not in the rectum. Transient passage of strains is an unlikely explanation for the detection of strains in multiple gut locations, given that *E. coli* were isolated from mucosal biopsies, and the loose adherent mucus was washed away before excision. The authors suggested that there may be *E. coli* site-specificity in the lower gut. Studying site-specificity in the gut may provide a greater understanding of the genotypic and phenotypic changes that occur as strains adapt to different niches.

The presence of *E. coli* in the human gut is remarkably consistent, even though it comprises just 0.01–1% of total gut anaerobes.^6,7^ *E. coli* can be divided into nine phylogroups: the major phylogroups (A, B1, B2 and D) and minor phylogroups (C, E, F, G and H).^6^ Strains belonging to B2 phylogroup are frequently detected in people living in developed regions, and phylogroup distribution is thought to be associated with food habits.^8,9^ Common human-associated B2 phylogroup strains which belong to sequence types (STs) ST73, ST95, and ST131, may cause extraintestinal infections, although these lineages have been recovered from asymptomatic humans.^10–13^

Based on the time spent in the host, *E. coli* can be transient or persistent. While transient strains can disappear from the host gut within days or weeks via fecal discharge, persistent strains can persist for months to years. Strains of the B2 phylogroup have been widely reported as persistent colonizers of the human gut.^3,14^

Evolution-driven changes in *E. coli* residing in the gut may occur randomly or through selection. Site-specific selective pressure on *E. coli* in the human gut may occur due to differences in motility, nutrient concentration, pH, and epithelial cell types in different gut regions, which may favor strains with certain properties.^15–17^ The evolution of *E. coli* genomes during residence within a host may also confer a survival advantage at a particular location.^18–20^ Mutations in *E. coli* chromosome(s) can accumulate in subsequent generations^20,21^ due to the influence of environmental stresses^22,23^ or the immune system^24^, resulting in genomic evolution. *E. coli* has been shown to persist in a host for up to six years, providing enough time for genomic evolution to occur.^25–27^ In humans, the most and second most abundant *E. coli* are likely members of the same phylogroup.^5^ New variants may appear due to a new colonization event or within-host evolution of a strain. Dixit *et al*^18^ estimated that new colonization events and within-host evolution contributed equally to the diversity of *E. coli* strains found in the gut. In that study, within-host evolution was defined as two or more strains of the same ST, isolated from the same individual and varying between 2 and 50 single nucleotide polymorphisms (SNPs). Given these findings, it can be hypothesized that *E. coli* undergoes genomic and phenotypic changes to adapt to different gut locations.

Several studies have reported tissue tropism of *E. coli* in the intestine, but most have focused on animal origins (e.g., bovine, porcine, and rodent) and pathogenic *E. coli*, such as Enterohemorrhagic *E*. *coli* (EHEC), Enterotoxigenic *E*. *coli* (ETEC), and Enteropathogenic *E*. *coli* (EPEC).^28–31^ In porcine studies, the recovery of *E. coli* phylogroups and genes for metabolite secretion was found to vary according to the gut regions.^32,33^ Some bacteriocin genes, such as colicin Ia/Ib, colicin B, and colicin M, were more frequently associated with strains isolated from the ileum than the duodenum, colon, or feces of pigs.^33^ Therefore, it is plausible that commensal *E. coli* site-specificity may also exist in the human gut. To the best of our knowledge, no study has determined whether commensal *E. coli* shows site-specificity in the human gut.

To do so, we used two similar *E. coli* strains (clone pairs) isolated from mucosal biopsies taken from two different locations in the lower gut (terminal ileum and rectum) of the same individual. We sought to identify differences in the genetic and phenotypic characteristics of the clone pairs that may account for site-specificity and within-host adaptation.

## Results

### Similarity of the clone pairs

Molecular profiling revealed that the strains belonging to each clone pair were highly similar. A comparison of the terminal ileum and rectum strains showed that approximately 97% of clone pairs (36/37) were similar according to sequence types (STs), O-, H-, and fimH types (Table 1). One pair (P−37) revealed different O-types, but identical ST, H-, and fimH types. The clone pairs were found to carry genes conferring resistance to several groups of antimicrobial drugs. Among the 37 clone pairs, 36 (97%) carried similar antimicrobial resistance genes and one pair (P−13) showed variation, where genes *aadA5_1* (aminoglycoside group), *dfrA17_1* (trimethoprim group), and *mph(A)_2* (MLS group: macrolide, lincosamide, and streptogramin) were present only in the terminal ileum strain (Figure S1).

**Table 1.** Basic molecular typing of Escherichia coli clone pairs according to ST, O-type, H-type and fimH type.

Clone pair	Location of strain collection	ST	O- type	H- type	fimH
P−1	Terminal ileum	ST95	O1	H7	fimH27
	Rectum	ST95	O1	H7	fimH27
P−2	Terminal ileum	ST95	O1	H7	fimH30
	Rectum	ST95	O1	H7	fimH30
P−3	Terminal ileum	ST95	O1	H7	fimH41
	Rectum	ST95	O1	H7	fimH41
P−4	Terminal ileum	ST95	O1	H7	fimH41
	Rectum	ST95	O1	H7	fimH41
P−5	Terminal ileum	ST95	O50/O2	H5	fimH526
	Rectum	ST95	O50/O2	H5	fimH526
P−6	Terminal ileum	ST95	O1	H7	fimH41
	Rectum	ST95	O1	H7	fimH41
P−7	Terminal ileum	ST95	O1	H7	fimH41
	Rectum	ST95	O1	H7	fimH41
P−8	Terminal ileum	ST95	O1	-	fimH41
	Rectum	ST95	O1	-	fimH41
P−9	Terminal ileum	ST95	O1	H7	fimH41
	Rectum	ST95	O1	H7	fimH41
P−10	Terminal ileum	ST95	O1	H7	fimH41
	Rectum	ST95	O1	H7	fimH41
P−11	Terminal ileum	ST131	O16	H5	fimH41
	Rectum	ST131	O16	H5	fimH41
P−12	Terminal ileum	ST131	O25	H4	fimH30
	Rectum	ST131	O25	H4	fimH30
P−13	Terminal ileum	ST131	O25	H4	fimH30
	Rectum	ST131	O25	H4	fimH30
P−14	Terminal ileum	ST131	O62	H4	fimH22
	Rectum	ST131	O62	H4	fimH22
P−15	Terminal ileum	ST131	O25	H4	fimH30
	Rectum	ST131	O25	H4	fimH30
P−16	Terminal ileum	ST131	O25	H4	fimH30
	Rectum	ST131	O25	H4	fimH30
P−17	Terminal ileum	ST131	O25	H4	fimH30
	Rectum	ST131	O25	H4	fimH30
P−18	Terminal ileum	ST131	O16	H5	fimH41
	Rectum	ST131	O16	H5	fimH41
P−19	Terminal ileum	ST80	O75	H7	fimH120
	Rectum	ST80	O75	H7	fimH120
P−20	Terminal ileum	ST80	O75	H7	fimH120
	Rectum	ST80	O75	H7	fimH120
P−21	Terminal ileum	ST80	O75	H7	fimH120
	Rectum	ST80	O75	H7	fimH120
P−22	Terminal ileum	ST80	O75	H7	fimH120
	Rectum	ST80	O75	H7	fimH120
P−23	Terminal ileum	ST73	O50/O2	H1	fimH41
	Rectum	ST73	O50/O2	H1	fimH41
P−24	Terminal ileum	ST73	O50/O2	H1	fimH41
	Rectum	ST73	O50/O2	H1	fimH41
P−25	Terminal ileum	ST73	-	H1	fimH10
	Rectum	ST73	-	H1	fimH10
P−26	Terminal ileum	ST569	O46, O134	H31	fimH5
	Rectum	ST569	O46, O134	H31	fimH5
P−27	Terminal ileum	ST569	O46, O134	H31	fimH5
	Rectum	ST569	O46, O134	H31	fimH5
P−28	Terminal ileum	ST569	O46, O134	H31	fimH5
	Rectum	ST569	O46, O134	H31	fimH5
P−29	Terminal ileum	ST372	O18, 018ac	H31	fimH9
	Rectum	ST372	O18, 018ac	H31	fimH9
P−30	Terminal ileum	ST372	O18, 018ac	H31	fimH9
	Rectum	ST372	O18, 018ac	H31	fimH9
P−31	Terminal ileum	ST14	O18, 018ac	H5	fimH27
	Rectum	ST14	O18, 018ac	H5	fimH27
P−32	Terminal ileum	ST91	O39	H4	fimH21
	Rectum	ST91	O39	H4	fimH21
P−33	Terminal ileum	ST491	O131	H45	fimH5
	Rectum	ST491	O131	H45	fimH5
P−34	Terminal ileum	ST550	O75	H5	fimH30
	Rectum	ST550	O75	H5	fimH30
P−35	Terminal ileum	ST636	O83	H7	fimH75
	Rectum	ST636	O83	H7	fimH75
P−36	Terminal ileum	ST1193	O18, 018ac	H5	fimH64
	Rectum	ST1193	O18, 018ac	H5	fimH64
P−37	Terminal ileum	ST11174	O107	H5	fimH5
	Rectum	ST11174	O107, O117	H5	fimH5

*P*-#, clone pairs; -, no hit found; The serotype gene sequences of both the terminal ileum and rectum strains of clone pair P−37 were checked to confirm whether they were incongruent.

**Figure 1.**
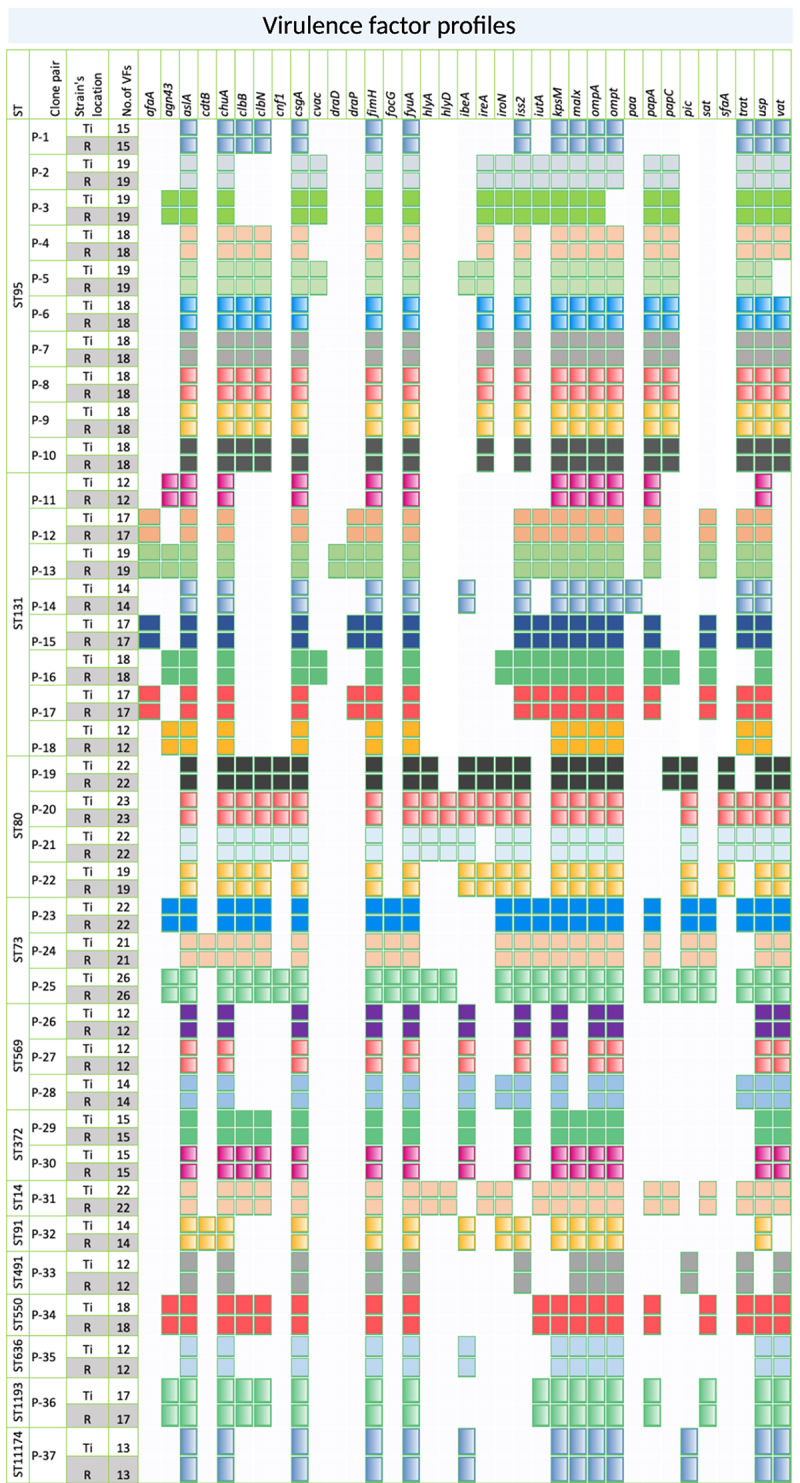
Profiling of virulence factor genes in *Escherichia coli* clone pairs. All clone pairs had identical virulence factor profiles. Blank and colored boxes indicate the absence and presence of the virulence factor genes, respectively. Ti, Terminal ileum; R, Rectum.

Plasmid analysis showed that 95% of the clone pairs carried similar plasmids; clone pairs P−7 and P−13 showed variation (Figure S2). Both the terminal ileum and rectum strains of P−13 carried the same number of plasmids (*N* = 8), where seven of the plasmids were the same and only one differed per strain. Col156_1 was present only in the terminal ileum and Col440II_1 was present only in the rectal strain. For virulence factor profiling, we considered only those genes frequently reported for the B2 phylogroup^34–38^, which included *afaA*, *agn43*, *aslA*, *cdtB*, *chuA*, *clbB*, *clbN*, *cnf1*, *csgA*, *cvac*, *draD*, *draP*, *fimH*, *focG*, *fyuA*, *hlyA*, *hlyD*, *ibeA*, *ireA*, *iroN*, *iss2*, *iutA*, *kpsM*, *malX*, *ompA*, *ompt*, *paa*, *papA*, *papC*, *pic*, *sat*, *sfaA*, *sfaS*, *trat*, *usp* and *vat*. The terminal ileum and rectum strains of all the clone pairs had identical virulence factor genes (Figure 1).

### Variation in mutations in the clone pairs

**Figure 2.**
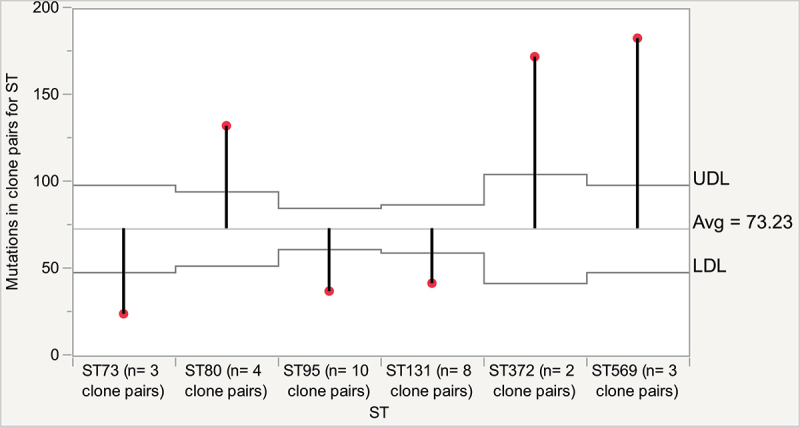
Analysis of means (ANOM) report for mutational differences between *Escherichia coli* clone pairs according to ST. Red tip indicates either significantly lower or higher mutational variation between clone pairs for each ST. UDL, upper decision limit; LDL, lower decision limit. Different upper and lower decision limits are due to variations in sample size of clone pairs for each ST. STs not connected by the same letter are significantly different. A, indicates that the number of mutational differences between clone pairs of ST73, ST95 and ST131 are not significantly different; B, indicates that the mutational differences in ST80 is significantly different from all other STs; C, indicates that the number of mutational differences in ST372 and ST569 are similar and significantly different from other STs. The statistical test was based on pair-wise comparisons of mutational differences for each ST using student’s t test and ordered difference report. STs containing only a single clone pair (ST14, ST91, ST491, ST550, ST636, ST1193, and ST11174) were not included in the analysis.

**Figure 3.**
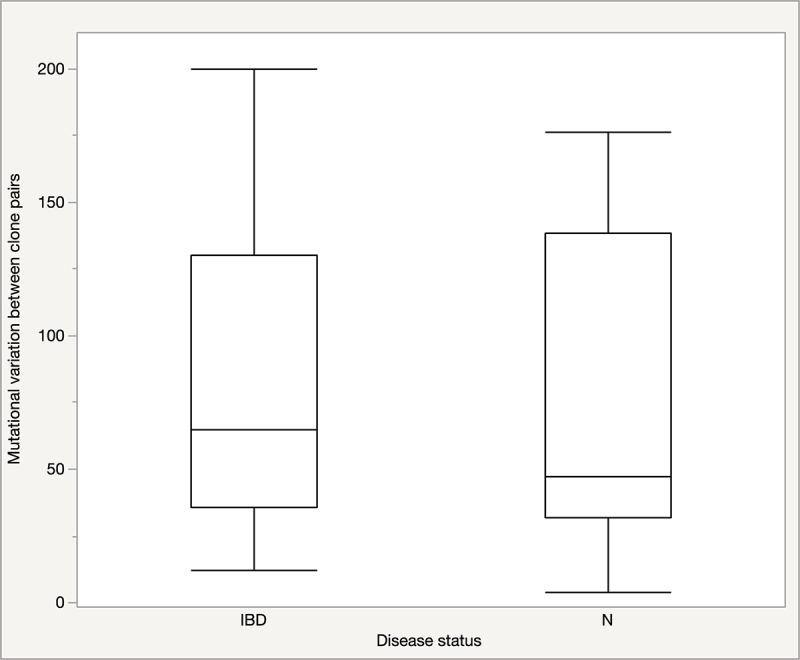
Distribution of mutational variation of clone pairs between patients with and without inflammatory bowel disease (IBD). IBD, individuals with Crohn’s disease (CD, eight clone pairs from seven individuals) and ulcerative colitis (UC, three clone pairs from three individuals); N, no disease (individuals without IBD or diarrheal conditions, 26 clone pairs from 24 individuals).

The Pathosystems Resource Integration Center (PATRIC, which operates under the Bacterial and Viral Bioinformatics Resource Center [BV-BRC; https://www.bv-brc.org/]) Variation Analysis provides genetic variation outputs in the form of single and multiple nucleotide polymorphisms (SNPs and MNPs), and insertions and deletions (Indels). Confidence scores are applied that correspond to calls such as synonymous mutations, non-synonymous mutations, and frameshifts. On average, each clone pair varied by 78 mutations (range: 4–200, Figure S3). SNPs were the most common type of genomic variation, followed by MNPs, and few Indels were observed. Analysis of means (ANOM) for STs represented by two or more clone pairs showed that the mean number of mutations between clone pairs did not vary within the human-associated STs, ST73, ST95, and ST131 (*p* > 0.05). However, the mean number of mutations for these human-associated STs was significantly lower (<50) than that for clone pairs belonging to non-human-associated STs, ST80, ST372, and ST569 (>130; *p* ≤ 0.05 [Figure 2]). We also determined whether the distribution of mutational variation between clone pairs differed according to the demographic characteristics of the individuals. The distribution of mutational variation did not vary according to the disease status (Figure 3), sex, or age of the individuals (*p* > 0.05).

### Analysis of mutational type, snpEFF-type, and snpEFF-impact

**Figure 4.**
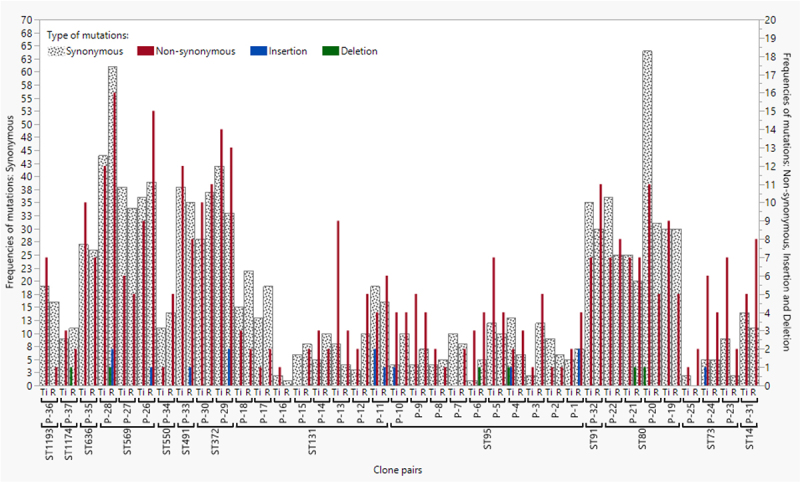
Frequency of types of mutations in *Escherichia coli* clone pairs across all STs. Y-axis at the left-hand side indicates the frequency of synonymous mutations, the right-hand side indicates the frequency of non-synonymous mutations and insertions and deletions. Statistical tests were performed for STs represented by two or more clone pairs only, and included: ST73, ST80, ST95, ST131, ST372 and ST569. Ti, Terminal ileum; R, Rectum.

PATRIC was used to provide additional information, such as mutational type, snpEFF-type, and snpEFF-impact of the mutations. Of the total mutations identified, 76% were synonymous, 23% were non-synonymous, and the remaining were indels (Figure 4). The terminal ileum and rectum strains of all STs did not vary significantly in carriage of synonymous or non-synonymous mutations. Synonymous mutations were excluded from the snpEFF-type analysis. The remaining mutations were missense variants, mutations in intergenic regions, frameshift variants, stop-gain variants, and stop-lost variants. Missense variants and mutations in intergenic regions were comparatively more common than those in other types. Although frameshift variants appeared in some pairs, there were very few stop-lost or stop-gain variants. However, neither the terminal ileum nor the rectum strains of any ST varied in terms of carrying missense variants, frameshift variants, or mutations in intergenic regions. Most of the non-synonymous mutational impacts were low (69%), followed by moderate (20%), modifier (10%), and high (1%) [data not shown]. Within an ST, neither the terminal ileum nor the rectum strains varied with respect to carrying high, moderate, or modifier impacts.

### Mutations in particular genes were not common to either terminal ileum or rectum strains

We sought to identify mutated gene(s) specific to the terminal ileum or rectum strains. The frequency with which a gene was associated with non-synonymous mutations was compared between the terminal ileum and rectum strains. Genes with the same frequency between the terminal ileum and rectum strains were excluded because they did not provide any distinguishing information. If mutations were associated with a gene exclusively related to either the terminal ileum or rectum strain, they were included in the analysis. From the gene lists, we excluded genes that were not likely to be core genes, such as genes encoding phage-associated proteins, plasmid conjugative transfer proteins (e.g., pilus assembly), transposases, mobile element proteins, insertion elements, iron uptake systems, and antimicrobial drug-associated proteins.

Comparing the terminal ileum and rectum strains overall and by ST, we did not find any gene(s) that were commonly associated with either the terminal ileum or rectum strains (Table S1a-S1m). We found numerous non-synonymous mutations associated with hypothetical proteins (HP). To distinguish hypothetical proteins, we recorded their upstream features (UF) and downstream features (DF) whenever possible. Although no signature gene(s) were identified, we observed that some genes appeared more commonly in the terminal ileum and rectum strains. Trehalose−6-phosphate hydrolase or genes related to trehalose appeared comparatively more commonly in the terminal ileum strains (Tables S1a, S1h, S1i, and S1l) and the uncharacterized protein YeeL in the rectum strains (Tables S1a, and S1k). In ST131, possible periplasmic thioredoxin (a specific protein name was not detected) was slightly more common in terminal ileum strains (Table S1b). In ST80, a hypothetical protein (no upstream or downstream features), in ST73 another hypothetical protein (downstream feature uncharacterized protein YfjI), and ST372 catalase KatE-intracellular protease were slightly more common in the terminal ileum strains (Tables S1c, S1d, and S1f). D-mannonate oxidoreductase and UPF0192 protein YfaS were found to be slightly more common in the rectal strains of ST569 (Table S1e).

### Metabolism differences between terminal ileum and rectum strains

**Figure 5.**
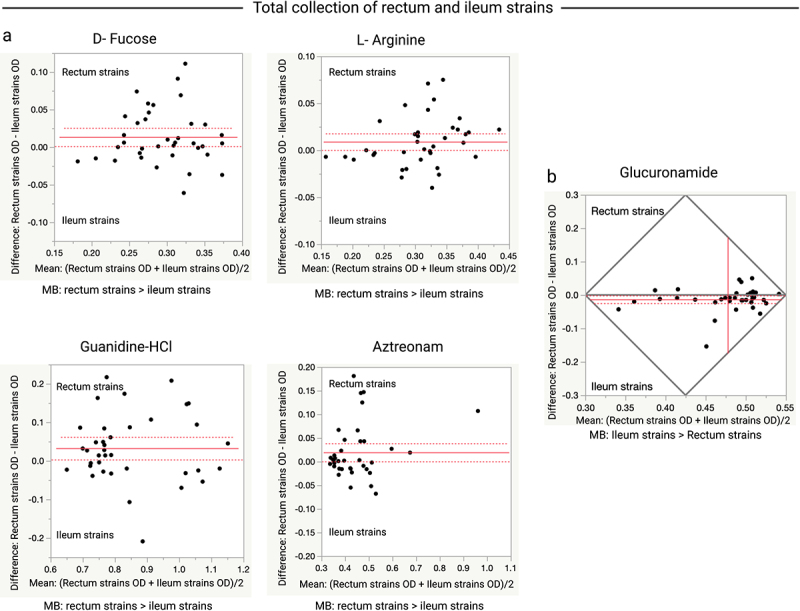
Comparison of OD values for the total collection terminal ileum (Ti) and rectum (R) clone pair strains of *E.* *coli* grown in various carbon sources. (a) Plots showing carbon sources where rectum strains showed higher metabolic activity than the ileum strains. (b) Plot showing that ileum strains had higher metabolic activity than rectum strains in glucuronamide. Data shown only for carbon sources where the ileum and rectum strains significantly varied in their metabolic activity. Paired t-tests were performed for this analysis. Each dot indicates the mean (x-axis) and difference (y-axis) for each clone pair. The red line or the intersected point of red lines indicates the mean difference (y-axis), the dashed red lines indicate the upper and lower confidence intervals (95%). MB, metabolism; >, indicates which strains (rectum or ileum) showed higher metabolic activity.

Biolog GENIII MicroPlates (CellBioscience) were used to biochemically profile the total collection of the terminal ileum and rectum strains. Clone pairs were included in the analysis if the terminal ileum and rectum strains showed evidence of a metabolic response in the presence of a specific carbon source but varied in their optical densities (ODs) [marked black color in Table S2]. As we did not have multiple assay replicates for each strain, we restricted our analysis to a comparison of the total collection of the terminal ileum and rectum strains and within ST comparisons of those that carried multiple clone pairs. The overall comparison showed that the rectum strains had higher ODs in D-fucose (*p* = 0.0317), L-arginine (*p* = 0.0428), guanidine HCl (*p* = 0.0303), and aztreonam (*p* = 0.0462) [Figure 5a], and the terminal ileum strains had higher ODs in glucuronamide (*p* = 0.0286) [Figure 5b].

**Figure 6.**
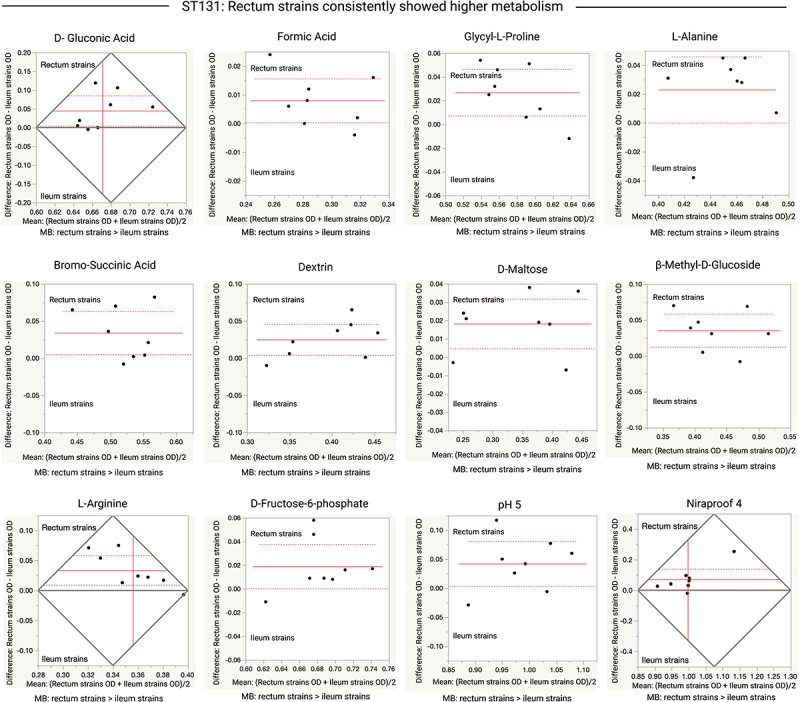
Comparison of OD values for the terminal ileum (Ti) and rectum (R) strains of *E.* *coli* ST131 clone pairs (eight clone pairs) grown in various carbon sources. Data shown only for carbon sources where the ileum and rectum strains significantly varied in their metabolic activity (D-Gluconic Acid [*p* = 0.0338], Formic Acid [*p* = 0.0424], Glycyl-L-Proline [*p* = 0.0143], L-Alanine [*p* = 0.0495], Bromo-Succinic Acid [*p* = 0.0276], Dextrin [*p* = 0.0257], D-Maltose [*p* = 0.0153], β-Methyl-D-Glucoside [*p* = 0.0083], L-Arginine [*p* = 0.0146], D-fructose −6-phosphate [*p* = 0.0469], pH 5 [*p* = 0.0361], Niraproof 4 [*p* = 0.0438]). Paired t-tests were performed for this analysis. Each dot indicates the mean (x-axis) and difference (y-axis) for each clone pair. The red line or the intersected point of red lines indicates the mean difference (y-axis), the dashed red lines indicate the upper and lower confidence intervals (95%). MB, metabolism; >, indicates which strains (rectum or ileum) showed higher metabolic activity.

**Figure 7.**
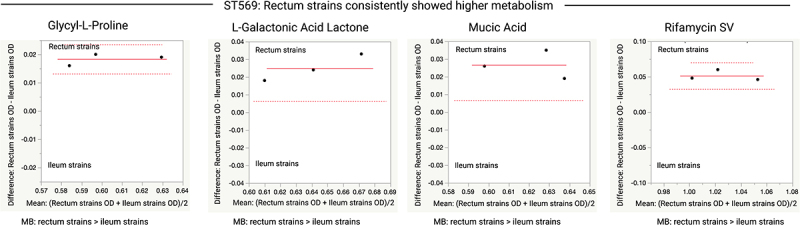
Comparison of OD values for the terminal ileum (Ti) and rectum (R) strains of *E.* *coli* ST569 clone pairs (three clone pairs) grown in various carbon sources. Data shown only for carbon sources where the ileum and rectum strains significantly varied in their metabolic activity (Glycyl-L-Proline [*p* = 0.0043], L-Galactonic Acid Lactone [*p* = 0.0291], Mucic Acid [*p* = 0.0289], and Rifamycin SV [*p* = 0.0072]). Paired t-tests were performed for this analysis. Each dot indicates the mean (x-axis) and difference (y-axis) for each clone pair. The red line indicates the mean difference (y-axis), the dashed red lines indicate the upper and lower confidence intervals (95%). MB, metabolism; >, indicates which strains (rectum or ileum) showed higher metabolic activity.

**Figure 8.**
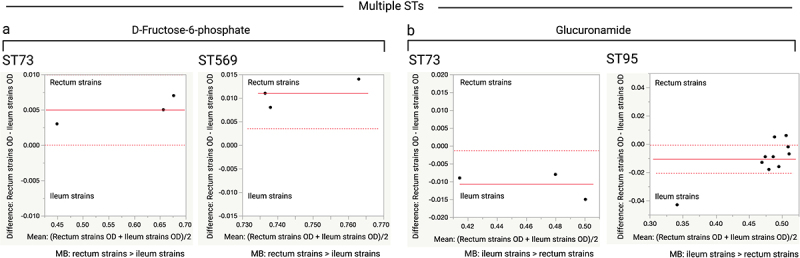
Comparison of OD values for the terminal ileum (Ti) and rectum (R) strains of *E.* *coli* of multiple STs (ST73: three clone pairs; ST569: three clone pairs, and ST95: ten clone pairs), when grown in the same carbon sources. (a) Plots showing that the rectum strains of multiple STs had higher metabolic activity than the ileum strains in D-fructose −6-phosphate (ST73 [*p* = 0.0494], and ST569 [*p* = 0.0239]). (b) Plots showing that the ileum strains of multiple STs had higher metabolic activity than the rectum strains in glucuronamide (ST73 [*p* = 0.0395], and ST95 [*p* = 0.0397]). Data shown only for carbon sources where the ileum and rectum strains significantly varied in their metabolic activity. Paired t-tests were performed for this analysis. Each dot indicates the mean (x-axis) and difference (y-axis) for each clone pair. The red line indicates the mean difference (y-axis), the dashed red lines indicate the upper and lower confidence intervals (95%). MB, metabolism; >, indicates which strains (rectum or ileum) showed higher metabolic activity.

In more than 75% of the observed variations, the rectal strains showed higher metabolic activity than the ileal strains. ST-wise comparisons revealed further variation between the terminal ileum and rectum strains with respect to the use of carbon sources and chemicals (*p* ≤ 0.05 or 0.01). In all ST131 and ST569 isolates, the rectal strains consistently showed higher metabolism than the ileal strains (Figures 6 and 7). The rectal strains of multiple STs showed higher metabolic activity than the ileal strain in the presence of D-fructose−6-phosphate (ST131 in Figure 6; ST73 and ST569 in Figure 8), glycyl-L-proline (ST131 in Figure 6 and ST569 in Figure 7), and formic acid (ST131 in Figure 6 and ST372 in Figure S4). In contrast, the terminal ileum strains of multiple STs showed higher metabolic activity than the rectal strains in the presence of glucuronamide (Figure 8b). The terminal ileum and rectum strains of ST131 did not show variation at pH 6.0, but did at pH 5.0; the rectum strains had higher ODs at pH 5.0 (Figure 6). The terminal ileum and rectum strains of ST80 varied when grown in 4% NaCl but not in 1% or 8% NaCl; rectum strains had higher ODs when grown in 4% NaCl (Figure S4).

### Growth rate variability between terminal ileum and rectum strains in different pH conditions

**Figure 9.**
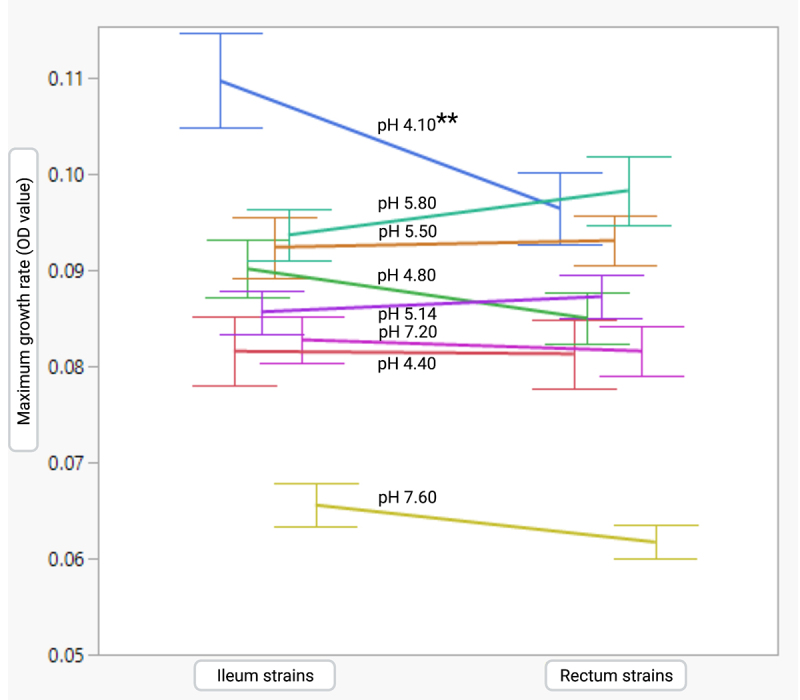
Overall comparison of maximum growth rates for the terminal ileum and rectum strains of *E.* *coli* clone pairs grown under different pH conditions. The analysis includes three technical replicates for each strain. Each error bar was produced using one standard deviation (±SD) from the mean. Paired t-tests were performed to determine the mean difference between the terminal ileum and rectum strains. **, *p* ≤ 0.01 otherwise *p* > 0.05.

**Figure 10.**
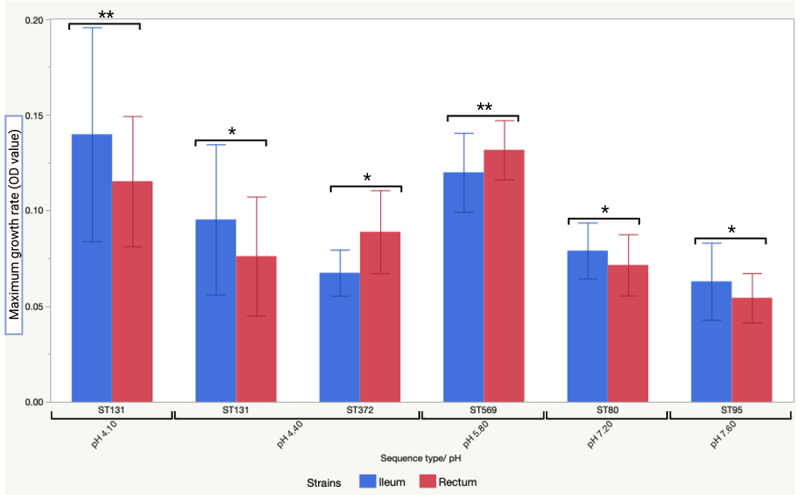
Maximum growth rate comparison between the terminal ileum and rectum strains of *E.* *coli* grown under different pH conditions across the STs. STs that had only one clone pair were not included in the analysis. Each error bar was produced using one standard deviation (±SD) from the mean. Paired t-tests were performed to determine the mean difference between the terminal ileum and rectum strains within an ST. *, *p* ≤ 0.05; **, *p* ≤ 0.01.

The overall comparison showed that the maximum growth rates of the terminal ileum and rectum strains varied significantly at pH 4.10; the terminal ileum strains had higher maximum growth rates than the rectum strains (Figure 9). The clone pairs did not vary significantly under other pH conditions. For some STs, the terminal ileum and rectum strains showed variations across multiple pH ranges (Figure 10). The maximum growth rates of the terminal ileum and rectum strains of ST131 showed significant variation at pH 4.10 and 4.40; the terminal ileum strains had higher growth rates under both pH conditions. Differences in growth rate were also observed for the terminal ileum and rectum strains belonging to ST80 and ST95 at different pH values. At pH 5.80, three STs showed significant variation between the terminal ileum and rectum strains. In all cases, the rectum strains showed significantly higher growth than the terminal ileum strains. At neutral pH (pH 7.20 and pH 7.60), two STs (ST80 and ST95) showed variation, with the terminal ileum strains showing higher growth rates. Each clone pair analysis showed that of the 37 clone pairs, a few clone pairs varied significantly according to the pH of their culture conditions (Figure S5). Considering only the pH conditions at which the maximum numbers of clone pairs showed variation, the terminal ileum strains showed a higher growth rate at pH 4.80, whereas the rectal strains showed higher growth rates at pH 5.80.

## Discussion

*E. coli* strains with identical MLVA profiles (“clone pairs”), were isolated from the terminal ileum and rectum and showed genomic variation (mainly SNPs), suggesting that clone pairs may not be truly identical at the genomic level. These variations were likely due to random mutations with a neutral effect. This study did not track genomic changes in strains over time, but identified genomic differences between strains isolated from the ileum and those isolated from the rectum. As members of the B2 phylogroup, it is likely that the clone pairs resided in the two gut locations for a longer period of time, which provides an opportunity for the clone pairs to undergo genomic differences.

Mutations and horizontal gene transfer events are two modes of bacterial evolution.^39–41^ A real-time evolutionary experiment carried out in mice showed that bacteriophage-mediated horizontal gene transfer exceeds mutations for rapid colonization of *E. coli* in the gut.^42^ As we did not find that the clone pairs varied in plasmid content, virulence factors, or antimicrobial resistance profiles, it is unlikely that the clone pairs were significantly affected by horizontal gene transfer events, and they were more likely to acquire mutations while residing in the human gut. In one study, multiple clones of *E. coli* ED1a were isolated from fecal samples from a human at three time points within one year; there was little evidence of phage integration or gain or loss of plasmids, but there was mutational variation between clones.^19^

The within-host evolution of *E. coli* has been reported previously.^18,26,27^ The genomic variation in clone pairs varies between human and non-human-associated STs. Clone pairs of STs that are more likely to be human-associated, such as, ST73, ST95, and ST131, showed fewer variations, suggesting that they are better adapted to the human gut. Clone pairs belonging to ST80, ST372, ST569, and ST636, which are less likely to be reported in human hosts^43–45^, showed greater variation. This suggests that these STs may have greater selective pressure to acquire more mutations to adapt to the human gut.^46,47^ However, it is difficult to ascertain a specific cause of this variation, as the human gut is a complex system and many mechanisms are simultaneously at play.^48^ It should be acknowledged that we did not compare a single pair isolated from the same site. Such a comparison could further strengthen our evidence of the genomic variation of clone pairs.

We analyzed non-synonymous gene mutations, especially the fimbrial and non-fimbrial factors, to identify the genetic signatures shared by the clone pairs. We did not detect any genes common to either the terminal ileum or the rectum isolates. This suggests that most non-synonymous mutations were accumulated neutrally. In a one-year *in vivo* study, mice fed a western diet and a chow diet showed a convergence effect on the lactose operon of *E. coli*, but the authors did not detect any molecular signature specific to either of the diets.^49^ The absence of a molecular signature was also observed in a longitudinal evolutionary study of an *E. coli* isolate that was dominant in the host for over one year.^19^ Tissue tropism is a feature of several *E. coli*, namely EPEC, EHEC and ETEC.^50–54^ ETEC causes diarrheal illnesses in infants, adults, and travelers, mainly in developing countries, and specifically colonizes and infects the small intestine.^31,55^ ETEC-associated adhesion or colonization factors include fibrillar proteins (CFA/1, CS1-CS7, CS14, CS17, CS21 and others)^31^, and other non-fimbrial adhesins (outer membrane protein Tia, autotransporter protein TibA, glycoprotein EtpA, pore-forming transport protein EtpB, and putative glycosyl transferase EtpC).^56^ We also found non-synonymous mutations associated with some fimbrial and membrane protein genes; however, they were not common to either the terminal ileum or rectum strains. The lack of a molecular marker that discriminates between the terminal ileum and rectum isolates may be due to the use of two genotypically similar B2 isolates that are resident colonizers of the gut. Alternatively, virulence factor genes alone may account for tissue tropism in pathogens, and the lack of virulence properties in commensal *E. coli* clone pairs meant that we did not observe a site-specific gene signature.^57,58^

Phenotypic characterization revealed the key observations that similar terminal ileum and rectum strains had different metabolic profiles. In more than 75% of the comparisons, the rectal strains showed higher metabolic activity than the terminal ileum strains. The double mucus layers that create a favorable bacterial niche in the colon may contribute to the rectal strains being better than the terminal ileum strains when using various carbon sources, such as d-fucose, and l-arginine.^59–62^ The terminal ileum strains showed higher metabolic activity than the rectum strains for one carbon source, glucuronamide, which is a type of hexose related to glucuronic acid. Glucuronic acid is involved in the formation of glucuronides in the liver, which are then transferred to the intestinal tract via biliary secretion.^63,64^ Among the bacterial communities, the genus *Escherichia* was comparatively more common than the other members of *Enterobacteriaceae* in duodenal fluid and bile samples.^65^ Enterohepatic circulation results in the uptake of bile salts in the distal small intestine, so that the colon is not exposed to bile salts. This may have resulted in the terminal ileum strains adapting to the utilization of glucuronamide better than the rectal strains.

The metabolic profile also showed that at least some STs (e.g., ST131 and ST569) may be better adapted to the rectum. For example, the clone pairs of ST131 showed metabolic variation in ten carbon sources and two chemicals; in all cases, the rectum strains showed higher metabolism than the terminal ileum strains. However, no metabolic signature was observed at the genomic level. This may be because the growth of clone pairs was more sensitive when grown in specific carbonaceous media (the observed variation in Biolog components), rather than simple lysogeny broth (LB) media, in which the strains were grown before DNA extraction for genome sequencing. Strains exhibiting greater metabolic potential may be important for strain colonization and adaptation in the intestine.^66^ Strains belonging to ST131 may have greater metabolic potential than non-ST131 strains^67^, and a superior capacity to colonize the mouse gut.^68^ However, no distinct metabolic properties of ST131 over non-ST131 have been reported in other studies.^69,70^ Nicolas-Chanoine *et al*^11^ suggested that the higher metabolic potential of ST131 may make it a more successful colonizers of the gut, which is required prior to its uropathogenicity. In our study, we found that clone pairs of ST131 showed the most phenotypic variation. The consistently higher metabolism of the rectum strains compared to the terminal ileum strains of ST131 suggests that strains of this ST may have better colonization and survival properties in the distal part of the gut, such as the rectum.

A possible limitation of this study is the use of draft genomes for the clone pairs. For the best output, we could have used the complete genome sequences of our strains and compared the clone pairs directly against each other. However, we compared each draft genome with a reference genome for the variation analysis. This approach allowed us to avoid any possible influence on mutational variation that may appear due to the use of draft genomes for clone pairs. Although, there were fewer examples of molecular trait variations between clone pairs, we confirmed any discrepancies by checking their coverage in the applicable contig. Some of the genomic variations found in the terminal ileum and rectum strains may have occurred before colonization in the gut or may have evolved in the laboratory before genomic DNA extraction. Studies have suggested the evolution of *E. coli* especially under long-term *in vitro* experimental conditions.^71,72^ We did not have evidence to determine the pre-colonization mutational effects of clone pairs. However, there were only two sub-culturing steps before DNA isolation, so it is unlikely that the clone pairs would have been significantly affected by *in vitro* evolution.

In conclusion, this study suggests that similar *E. coli* isolates from different gut locations are not truly similar at the genomic or phenotypic level. We found that clone pairs belonging to STs more frequently isolated from the human gut had less genomic variation, suggesting that their evolutionary history makes them better adapted to the gut. The lack of specific genetic signatures between the terminal ileum and rectum strains suggests that the observed variations are not due to site-specificity but rather because of the neutral evolution of strains. While the use of two genetically similar strains may have prevented us from identifying site-specific gene signatures of clone pairs, this study provides evidence that clone pairs isolated from different gut locations are genetically divergent. The site-specificity of *E*. *coli* is better observed from phenotypic studies that showed differences between clone pairs isolated from different gut locations, including in the metabolism of different carbon sources and growth at different pH, suggesting that strains adapt to different gut regions. Overall, this study showed that a strain’s genomic and phenotypic properties can be influenced by gut location, suggesting *E. coli* may be site-specific or show a level of plasticity when colonizing different gut regions.

## Materials and methods

### Ethics approval

This study was approved by the ACT (Australian Capital Territory) Health Research Ethics Committee (ETH.5.07.464) and the ANU (Australian National University) Human Research Ethics Committee (2012/596), ACT, Australia.

### Defining Escherichia coli clone pairs

In this study, a clone pair represents two *E. coli* isolates with the same MLVA gel electrophoresis profile belonging to the B2 phylogroup, where one isolate was obtained from the terminal ileum and the other from the rectum of the same individual (Figure S6). Given the dominance and colonization persistence of B2 phylogroup strains in the human gut, strain selection was restricted to B2 phylogroup strains.

### Collection of clone pairs

A total of 37 clone pairs isolated from 34 individuals were selected for this study (Table S3). Individual gut mucosal biopsies were collected by the designated doctors at the Canberra Hospital, ACT, Australia. Individuals provided consent prior to specimen collection for this study. The individuals included both females (*N* = 17), and males (*N* = 17), ranging in age from 21 to 78 years old. Of the 37 clone pairs, 30 were collected in 2019 for this study, and seven were collected from a previous study^5^, in which strain collection was performed between 2010 and 2011.

### Whole genome sequencing

DNA was isolated using an Isolate II Genomic DNA Kit (Bioline, Cat. No. BIO− 52067). DNA libraries were prepared using the Illumina Nextera DNA Flex Library Prep (96 samples: Illumina, Cat. No. 20018705) and IDT® for Illumina Nextera DNA Unique Dual Indexes Set A (96 indices, 96 samples: Illumina, Cat. No. 20027213), according to the manufacturer’s protocol. The library was sequenced using the MiSeq system (Illumina) along with the MiSeq Reagent Kit V3 (600 cycles, 300 bp paired end). The Biomolecular Resource Facility (BRF), Australian National University, prepared the library and performed the sequencing.

### Microbial genome assembly and phylogroup confirmation

SPAdes in EnteroBase was used to assemble the sequence^73^ and assembled genomes were aligned to a reference genome (S88) in Mauve.^74^ We confirmed the phylogroups of all strains using ClermonTyping.^75^

### Basic molecular profiling of clone pairs

The ST of each strain was determined using the MLST scheme of the EnteroBase (http://enterobase.warwick.ac.uk/) database.^76^ For serotyping (O-, H- type) and fimbriae typing (fimH type), SeroTypeFinder^77^ and FimTyper^78^ on the Center for Genomic Epidemiology (CGE) were used (http://www.genomicepidemiology.org/), respectively. CGE’s ResFinder 4.1 tool^79^ was used to detect the antimicrobial resistance genes, PlasmidFinder 2.1^80^ to detect plasmids, and VirulenceFinder 2.0^81^ for virulence factor profiling.

### Genome annotation and reference strain selection

We annotated the assembled draft genomes in PATRIC (now BV-BRC) using RASTtk, available at https://www.patricbrc.org/app/Annotation.^82^ We determined the ST of all strains using the MLST EnteroBase database. We identified B2 reference strains (complete genomes) specific to each ST in the National Center for Biotechnology Information (NCBI) database and downloaded their genomes, including plasmids, whenever applicable. For plasmid(s)-bearing genomes, we added the nucleotide sequences of plasmid(s) to the chromosomal nucleotide sequences. We then annotated all the downloaded reference strains in PATRIC. Phylogenetic analysis was conducted by comparing all the strains with the reference genomes. When we did not find a reference strain for an ST, we used the reference strain of a closely related ST. A list of the reference genomes for each ST is provided in Table S4.

### Identification of mutations

We used the Variation Analysis tool in PATRIC to analyze mutational variation between the clone pairs.^83^ We separately analyzed the terminal ileum and rectum strains of each clone pair against the reference strain of the same ST. The read files of a desired bacterial strain were uploaded in the “paired read library” and the genome of a reference strain was uploaded in the “target genome”. We selected “BWA-mem-strict” as the aligner and “Freebayes” as the SNP caller. We retrieved the “all.var.tsv” files, which contained variation information for each strain (terminal ileum/rectum strains) against the reference strain. For determining mutations between similar strains of each clone pair, the “all.var.tsv” files of the terminal ileum and rectum strains were joined together in JMP version 15 software. Then, the terminal ileum strain was compared with the rectum strain, and the contig and position for matched and non-matched mutations with the reference strain were obtained. Using the conditional edit formula, the matched mutations between terminal ileum and rectum strains against the reference strain were denoted as ‘0’ and non-matched were denoted as ‘1.’ We selected only the non-matched mutations as the expected variation between the clone pairs (similar terminal ileum and rectum strains). We then checked the quality scores of all respective mutations. We performed quality control of the sequence variations at greater scores. We removed the mutations that were below the 90% quantile and marked the remaining mutations between clone pairs. As PATRIC also provides annotation of the mutations, we determined the mutational types, snpEFF_type, snpEFF_impact, and non-synonymous associated gene functions.

### Metabolic profiling of clone pairs

Biolog GENIII MicroPlates (CellBioscience, Cat. No. B 1030) were used to assess the ability of each strain to use 71 different carbon sources and their performance in 23 chemical-sensitivity reactions (Table S2). Freezer stocks were grown overnight on LB agar plates at 35°C. A single colony was picked and thoroughly mixed with the GENIII inoculation fluid (Cat. No. B72401). Then, 100 µL of the inoculated GENIII fluid was transferred to each well of a Biolog GENIII MicroPlate and incubated at 35°C for 24 h under static conditions. To measure the endpoint growth, turbidity and color intensity of the wells, the OD value was measured at 640 nm using a microplate reader (PowerWave_X_ 340, BIO-TEK Instruments Inc.). The assay was performed once for each strain.

### Determination of maximum growth rate in different pH conditions

The strains were grown overnight in LB broth at 35°C with shaking at 150 rpm. Then, 5 µL of overnight culture was inoculated in 190 µL Davis Minimal Media at various pH levels (pH 4.10, 4.40, 4.80, 5.14, 5.50, 5.80, 7.20, and 7.60) in a polystyrene round-bottom 96-well plate (Greiner Bio-one). After inoculation, we inserted the plate into a microplate reader (PowerWave_X_ 340, BIO-TEK Instruments Inc.) and set the OD value at 600 nm. To maintain the exponential phase, we incubated the cells for 18 h at 35°C and set the reader in kinetic mode, so that the plate was vibrated (mixed) for 5-second before every 10-minute OD reading.

### Statistical analysis

Statistical tests were performed using the software package JMP version 15 (SAS Institute Inc., 2019). The growth rate and maximum growth rate were calculated using Excel. Student’s t-test was used to compare the mean mutations between the two different STs. We used paired t-tests for determining mutational variation between clone pairs within a ST. Paired t-tests were used to compare means between the terminal ileum and rectum strains in phenotypic assays. Statistical significance was set at a 95% confidence interval (*p* ≤ 0.05).

## Supplementary Material

Supplemental Material

## Data Availability

The genome sequences of *Escherichia coli* strains used in this study were deposited and are available in EnteroBase (https://enterobase.warwick.ac.uk/).
