# Supplementary material for: Comparative genomics and phenotypic studies to determine site-specificity of Escherichia coli in the lower gastrointestinal tract of humans
Source: Gut Microbes. 2023 Jun 20;15(1):2223332. doi: 10.1080/19490976.2023.2223332 (PMC10286668; doi:10.1080/19490976.2023.2223332)
Supplement: Supplemental Material [file KGMI_A_2223332_SM1211.docx]

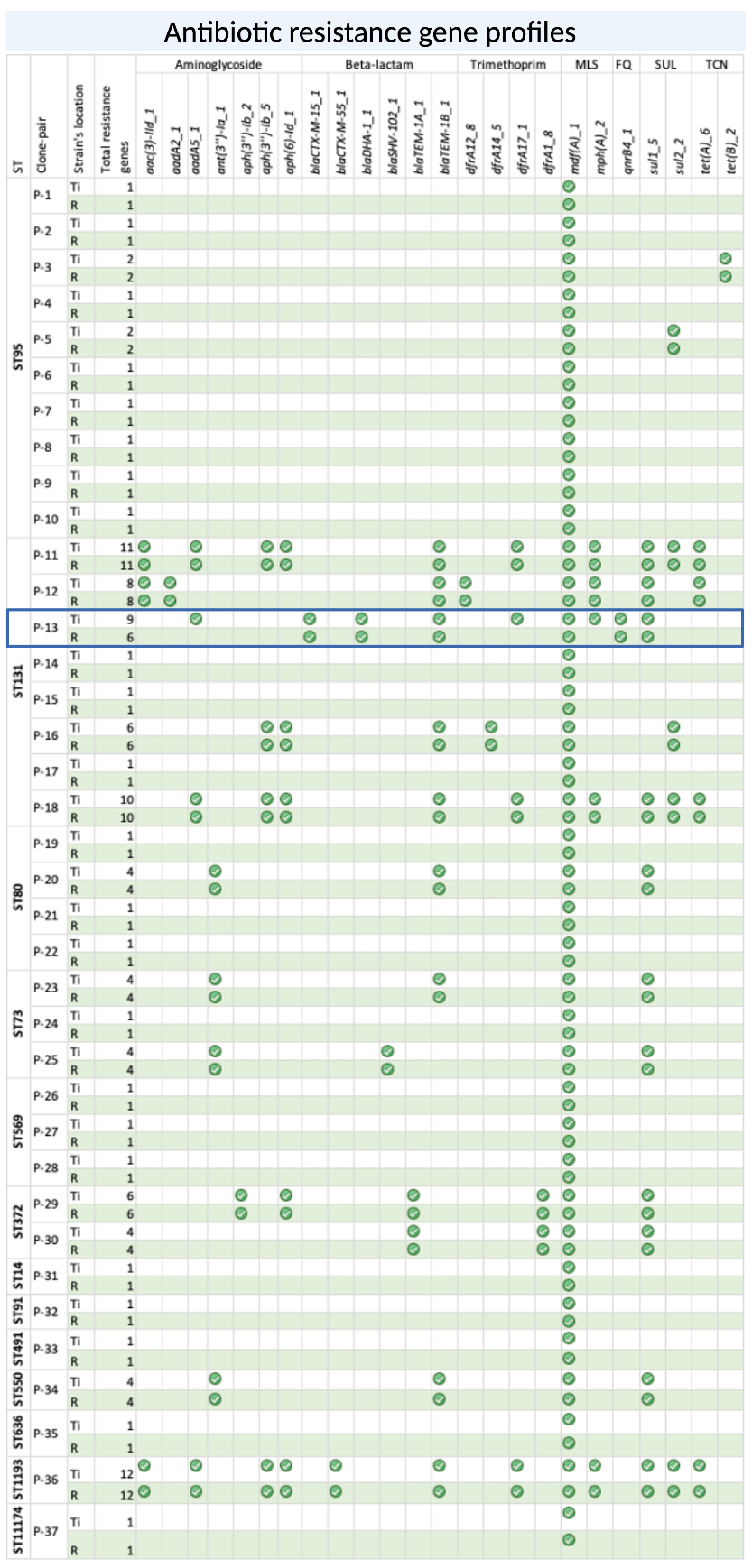


Figure S1. Antimicrobial resistance gene profiles of *Escherichia coli* clone pairs. Blank indicates susceptibility and green with tick inside indicates resistance to the antibiotics. Blue box indicates that the pair had incongruent antibiotic resistance gene profile of *aadA5_1*, *dfrA17_1*, and *mph(A)_2*. MLS, macrolide, lincosamide and streptogramin; FQ, fluoroquinolone; SUL, sulfonamide; TCN, tetracycline; Ti, Terminal ileum; R, Rectum.


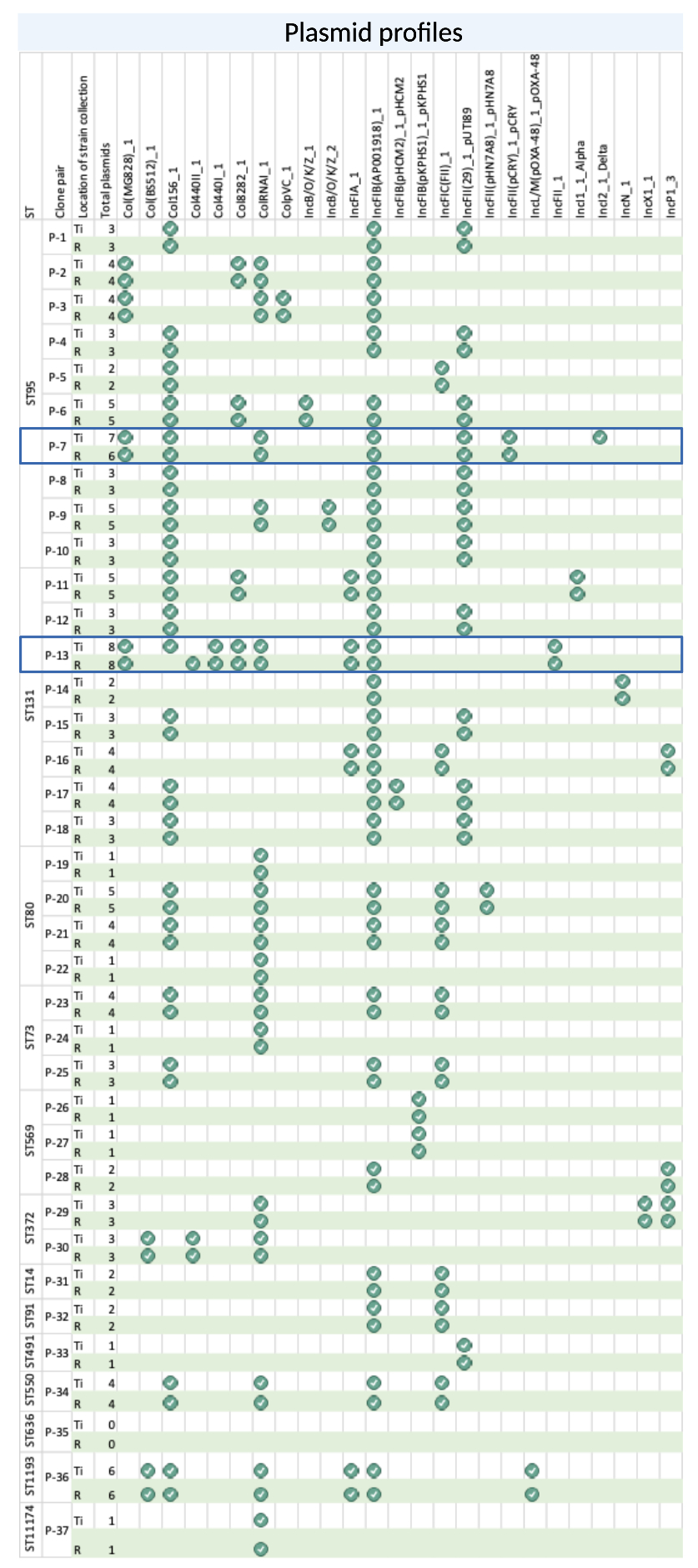


Figure S2. Plasmid distribution in *Escherichia coli* clone pairs. Blank indicates absence of plasmids and green with tick indicates presence of plasmids. Blue boxes, indicates that the pair-7 had incongruent plasmid profile of IncI2_1_Delta and pair-13 had incongruent plasmid profile of Col156_1 and Col440II_1. Ti, Terminal ileum; R, Rectum.


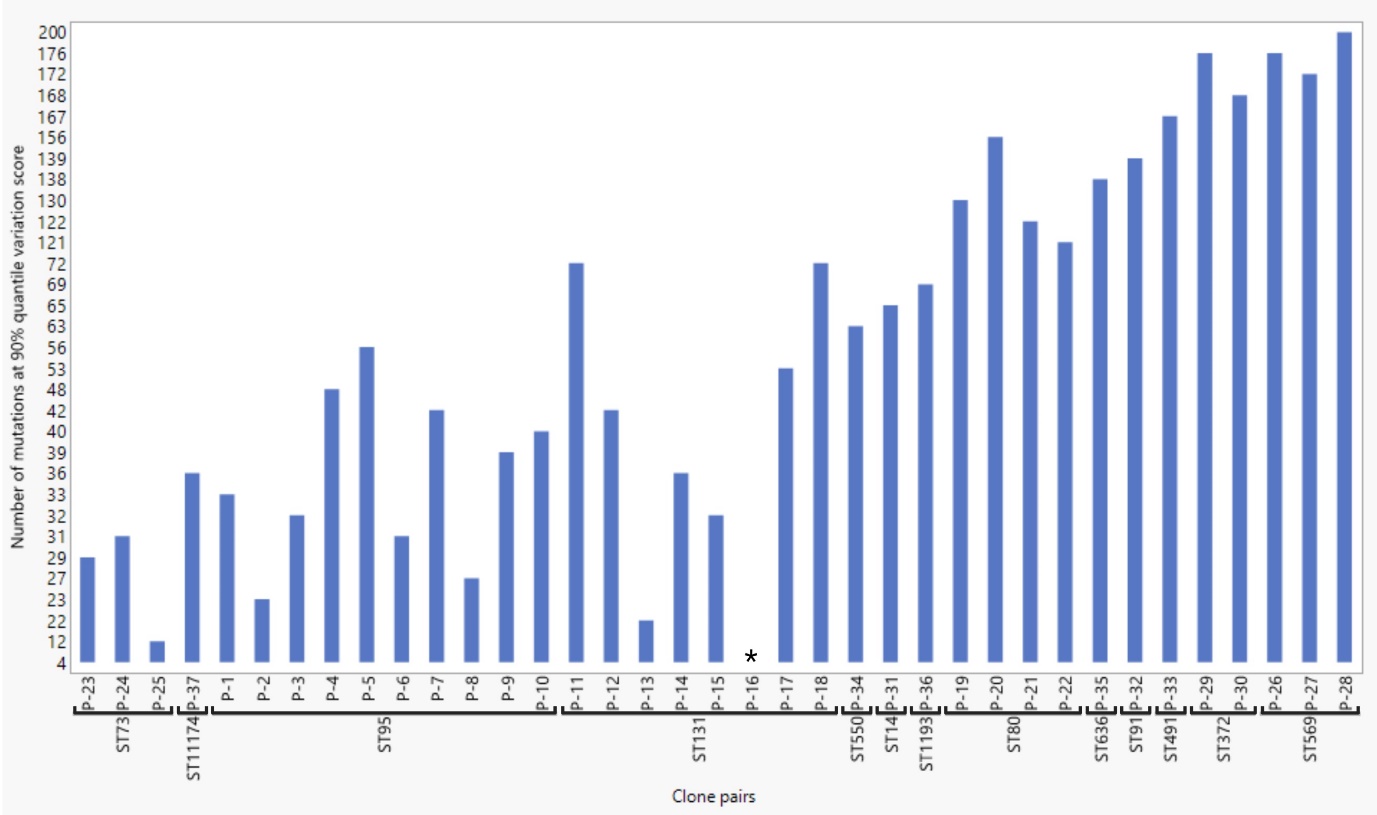


**Figure S3.** Representation of mutations observed in *Escherichia coli* clone pairs. The distribution of mutational variation in clone pairs is grouped according to ST. The ‘X’ axis represents clone-pairs and ‘Y’ axis represents the numerical value of mutations in each pair. ‘*’ in P-16, the terminal ileum and rectum strains varied by 4 mutations.

Table S1. Non-synonymous associated gene functions in the terminal ileum and rectum strains (clone pairs) of *Escherichia coli* ST95 (a), ST131 (b), ST80 (c), ST73 (d), ST569 (e), ST372 (f), ST14 (g), ST91 (h), ST491 (i), ST550 (j), ST636 (k), ST1193 (l) and ST11174 (m).

**a- ST95**

| **Gene associated functions** | **Gut location** | **Frequency** |
| --- | --- | --- |
| Blue copper oxidase CueO precursor | Ileum | 1 |
| Clamp-binding protein paralog | Ileum | 1 |
| Free methionine-(Reductase)-sulfoxide Reductase, contains GAF domain | Ileum | 1 |
| Glucans biosynthesis glucosyltransferase H | Ileum | 1 |
| Histidinol-phosphate aminotransferase (EC 2.6.1.9) | Ileum | 1 |
| L, D-transpeptidase | Ileum | 1 |
| Nicotinate-nucleotide--dimethyl benzimidazole phosphoribosyl transferase (EC 2.4.2.21) | Ileum | 1 |
| Putative heme iron utilization protein | Ileum | 1 |
| Thymidine phosphorylase (EC 2.4.2.4) | Ileum | 1 |
| Uncharacterized protein YjiL | Ileum | 1 |
| YVTN beta-propeller repeat-containing protein | Ileum | 1 |
| Trehalose-6-phosphate hydrolase (EC 3.2.1.93) | Ileum | 6 |
|  | Rectum | 2 |
| Antirestriction protein klcA | Rectum | 1 |
| Colanic acid biosynthesis acetyltransferase WcaF | Rectum | 1 |
| Colanic acid biosynthesis glycosyl transferase WcaI | Rectum | 1 |
| Electron transfer flavoprotein-quinone oxidoreductase YgcN | Rectum | 1 |
| HP-UF- Cell division inhibition protein DicB | Rectum | 1 |
| HP-DF-Type 1 fimbriae regulatory protein FimB | Rectum | 1 |
| Mg (2+) transport ATPase, P-type (EC 3.6.3.2) | Rectum | 1 |
| NAD(P)H dehydrogenase (quinone) 2 (EC 1.6.5.2) | Rectum | 1 |
| Probable 9-O-acetyl-N-acetylneuraminic acid deacetylase | Rectum | 1 |
| Ribonuclease E (EC 3.1.26.12) | Rectum | 1 |
| Uncharacterized inner membrane protein YjiN | Rectum | 1 |
| Uncharacterized protein YeeL | Rectum | 2 |
| Uncharacterized protein YhjG | Rectum | 1 |

HP, hypothetical protein; UF, upstream feature; DF, downstream feature.

b- ST131

| **Gene associated functions** | **Gut location** | **Frequency** |
| --- | --- | --- |
| Acetaldehyde dehydrogenase (EC 1.2.1.10), ethanolamine utilization cluster | Ileum | 1 |
| c-di-GMP phosphodiesterase (EC 3.1.4.52) | Ileum | 1 |
| Efflux ABC transporter, permease/ATP-binding protein MdlA | Ileum | 1 |
| Glucose-1-phosphatase (EC 3.1.3.10) | Ileum | 1 |
| HP-DF-Sodium-dependent phosphate transporter | Ileum | 1 |
| HP-UF--mobile element protein | Ileum | 1 |
| HP-no UF or DF | Ileum | 1 |
| Malate synthase G (EC 2.3.3.9) | Ileum | 1 |
| Multimodular transpeptidase-transglycosylase (EC 2.4.1.129) (EC 3.4) | Ileum | 1 |
| Ornithine decarboxylase (EC 4.1.1.17) | Ileum | 1 |
| Possible periplasmic thiredoxin | Ileum | 2 |
| Protein FimG (regulates length and adhesion of type 1 fimbriae) | Ileum | 1 |
| Protease VII (Omptin) precursor (EC 3.4.23.49)  @ Outer membrane protease OmpT | Ileum | 1 |
| Uncharacterized protein YaiT | Ileum | 1 |
| Uncharacterized transporter YeeO | Ileum | 1 |
|  | Rectum | 2 |
| Dienelactone hydrolase family protein | Rectum | 1 |
| Glucose dehydrogenase, PQQ-dependent (EC 1.1.5.2) | Rectum | 1 |
| HP-DF-Phage tail formation protein GpD | Rectum | 1 |
| HP-associated with serine palmitoyl transferase | Rectum | 1 |
| Lipase, GDXG family | Rectum | 1 |
| Lipid A biosynthesis lauroyl acyltransferase (EC 2.3.1.241) | Rectum | 1 |
| Methylmalonyl-CoA mutase (EC 5.4.99.2) | Rectum | 1 |
| Oxygen-independent coproporphyrinogen-III oxidase-like protein YggW | Rectum | 1 |
| UDP-glucose:(glucosyl) lipopolysaccharide-alpha-1,2-glucosyltransferase (EC 2.4.1.58) | Rectum | 1 |

HP, hypothetical protein; UF, upstream feature; DF, downstream feature.

c- ST80

| **Gene associated functions** | **Gut location** | **Frequency** |
| --- | --- | --- |
| [4Fe-4S] cluster assembly scaffold protein Mrp | Ileum | 1 |
| 5'-nucleotidase (EC 3.1.3.5) | Ileum | 1 |
| Anaerobic glycerol-3-phosphate dehydrogenase subunit B (EC 1.1.5.3) | Ileum | 1 |
| Autonomous glycyl radical cofactor | Ileum | 1 |
| Cellulose synthase catalytic subunit [UDP-forming] (EC 2.4.1.12) | Ileum | 1 |
| DNA polymerase IV (EC 2.7.7.7) | Ileum | 1 |
| Exodeoxyribonuclease I (EC 3.1.11.1) | Ileum | 1 |
| Glutamate synthetase [NADPH] small chain (EC 1.4.1.13) | Ileum | 1 |
| Hexose-phosphate-uptake two-component transcriptional response regulator UhpA | Ileum | 1 |
| HP- no UF or DF | Ileum | 2 |
| Inner membrane component of TAM transport system | Ileum | 1 |
| Inner membrane protein YcfT | Ileum | 1 |
| Outer membrane porin for chitooligosaccharides ChiP | Ileum | 1 |
| Phosphoglycerate transport system sensor protein PgtB | Ileum | 1 |
| Phosphoglycerate transporter protein PgtP | Ileum | 1 |
| Probable tonB-dependent receptor yncD precursor | Ileum | 1 |
| Protein ViaA | Ileum | 1 |
| Protein SprT | Ileum | 1 |
| Putative iron compound receptor | Ileum | 1 |
| Putative oxidoreductase | Ileum | 1 |
| Sugar phosphatase YidA (EC 3.1.3.23) | Ileum | 1 |
| Uncharacterized protein YrhB | Ileum | 1 |
| Uroporphyrinogen-III synthase (EC 4.2.1.75) | Ileum | 1 |
| ATP-dependent helicase HrpB | Rectum | 1 |
| CFA/I fimbrial subunit C usher protein | Rectum | 1 |
| Enterobactin esterase | Rectum | 1 |
| Isoleucyl-tRNA synthetase (EC 6.1.1.5) | Rectum | 1 |
| L-carnitine/gamma-butyrobetaine antiporter | Rectum | 1 |
|  |  |  |
| Miniconductance mechanosensitive channel YbdG | Rectum | 1 |
| Outer membrane component of TAM transport system | Rectum | 1 |
| Phosphate ABC transporter, permease protein PstA (TC 3.A.1.7.1) | Rectum | 1 |
| Predicted regulator of STY3230 transporter operon | Rectum | 1 |
| Protein FimF (regulates length and adhesion of type 1 fimbriae) | Rectum | 1 |
| Protein with similarity to RtcB | Rectum | 1 |
| PTS, beta-galactosidase IIB comp / PTS, beta-galactosidase IIC comp / PTS, beta-galactosidase IIA comp | Rectum | 1 |
| Selenite- and tellurite-inducible protein TsgA | Rectum | 1 |
| Trna-5-carboxymethylaminomethyl-2-thiouridine (34) synthesis protein MnmE | Rectum | 1 |
| Transcriptional regulator PhnF | Rectum | 1 |
| Uncharacterized [4Fe4S] protein YgfT | Rectum | 1 |
| VgrG protein | Rectum | 1 |

HP, hypothetical protein; UF, upstream feature; DF, downstream feature.

**d- ST73**

| **Gene associated functions** | **Gut location** | **Frequency** |
| --- | --- | --- |
| 3-oxoacyl-[ACP] synthase (EC 2.3.1.41) FabV like | Ileum | 1 |
| Glycolate dehydrogenase (EC 1.1.99.14), subunit GlcD | Ileum | 1 |
| Glyoxylate carboligase (EC 4.1.1.47) | Ileum | 1 |
| Invasin | Ileum | 1 |
| putative secreted protein | Ileum | 1 |
| Uncharacterized protein YehM | Ileum | 1 |
| Xaa-Pro dipeptidase PepQ (EC 3.4.13.9) | Ileum | 1 |
| YehP, CoxE-like von Willebrand factor type A- domain-containing protein | Ileum | 1 |
| HP-DF-Uncharacterized protein YfjI | Ileum | 3 |
|  | Rectum | 1 |
| Membrane protease family protein y2843 | Rectum | 1 |
| Per-activated serine protease autotransporter enterotoxin EspC / autotransporter domain, T5aSS type secretion | Rectum | 1 |
| Putative alanine/glycine transport protein | Rectum | 1 |
| Pyruvate dehydrogenase (quinone) (EC 1.2.5.1) | Rectum | 1 |
| UPF0225 protein YchJ | Rectum | 1 |

HP, hypothetical protein; DF, downstream feature.

e- ST569

| **Gene associated functions** | **Gut location** | **Frequency** |
| --- | --- | --- |
| 2-acyl-GPE-acyltransferase (EC 2.3.1.40)/ Acyl-[acyl-carrier-protein] synthase (EC 6.2.1.20) (EC 2.3.1.40) / (EC 6.2.1.20) | Ileum | 1 |
| ABC transporter protein IroC | Ileum | 1 |
| Chitinase (EC 3.2.1.14) | Ileum | 1 |
| DNA recombination-dependent growth factor RdgC | Ileum | 1 |
| Evolved beta-D-galactosidase transcriptional repressor | Ileum | 1 |
| Glutamine-dependent 2-keto-4-methylthiobutyrate transaminase | Ileum | 1 |
| HP-UF-Dipeptide ABC transporter, substrate binding protein DppA (TC 3.A.1.5.2) @ Dipeptide chemoreceptor and DF-Dipeptide ABC transporter, permease protein DppB (TC 3.A.1.5.2) | Ileum | 1 |
| N-acetyltransferase ElaA | Ileum | 1 |
| RatA homolog | Ileum | 1 |
| Tetraacyldisaccharide 4'-kinase (EC 2.7.1.130) | Ileum | 1 |
| Uncharacterized protein YciQ | Ileum | 1 |
| Uncharacterized tRNA/rRNA methyltransferase LasT | Ileum | 1 |
| Uncharacterized transporter MdtD of major facilitator superfamily (MFS) | Ileum | 1 |
| Uncharacterized lipoprotein yaeF precursor | Ileum | 1 |
| YjfP protein | Ileum | 1 |
| Uncharacterized protein YihF | Ileum | 1 |
| 3-ketoacyl-CoA thiolase (EC 2.3.1.16) | Rectum | 1 |
| Acetyl-CoA acetyltransferase (EC 2.3.1.9) | Rectum | 1 |
| Aspartate carbamoyl transferase (EC 2.1.3.2) | Rectum | 1 |
| beta-galactosidase (EC 3.2.1.23) | Rectum | 1 |
| Cell division protein ZapE | Rectum | 1 |
| copper sensing two-component system response regulator- CpxR | Rectum | 1 |
| D-mannonate oxidoreductase (EC 1.1.1.57) | Rectum | 2 |
| Electron transfer flavoprotein, beta subunit YgcR | Rectum | 1 |
| Exonuclease SbcC | Rectum | 1 |
| Flagellar regulator flk | Rectum | 1 |
| Formate hydrogenlyase regulatory protein HycA | Rectum | 1 |
| Glycerate kinase (EC 2.7.1.31) | Rectum | 1 |
| Inner membrane component of TAM transport system | Rectum | 1 |
| LysR family transcriptional regulator YnfL | Rectum | 1 |
| Membrane protein | Rectum | 1 |
| Nitrogen regulation protein NR(I), GlnG | Rectum | 1 |
| Oxygen-independent coproporphyrinogen-III oxidase-like protein YggW | Rectum | 1 |
| Ribosomal protein S12p Asp88 (E. coli) methylthiotransferase (EC 2.8.4.4) | Rectum | 1 |
| tRNA-dihydrouridine (16) synthase | Rectum | 1 |
| TDP-N-acetylfucosamine: lipid II N-acetylfucosaminyltransferase (EC 2.4.1.325) | Rectum | 1 |
| Trans-aconitate 2-methyltransferase (EC 2.1.1.144) | Rectum | 1 |
| Transcriptional regulator GadX, AraC family | Rectum | 1 |
| Uncharacterized protein YidR | Rectum | 1 |
| Uncharacterized oxidoreductase YgjR | Rectum | 1 |
| Uncharacterized protein YmdF | Rectum | 1 |
| Uncharacterized protein YrhB | Rectum | 1 |
| UPF0192 protein YfaS | Rectum | 2 |

HP, hypothetical protein; UF, upstream feature; DF, downstream feature.

f- ST372

| **Gene associated functions** | **Gut location** | **Frequency** |
| --- | --- | --- |
| 2-oxoglutarate dehydrogenase E1 component (EC 1.2.4.2) | Ileum | 1 |
| 5'-nucleotidase SurE (EC 3.1.3.5) @ Exopolyphosphatase (EC 3.6.1.11) | Ileum | 1 |
| ABC transporter, ATP-binding protein (cluster 5, nickel/peptides/opines) | Ileum | 1 |
| ABC-type siderophore export system, fused ATPase and permease components | Ileum | 1 |
| Aerobic glycerol-3-phosphate dehydrogenase (EC 1.1.5.3) | Ileum | 1 |
| c-di-GMP phosphodiesterase (EC 3.1.4.52) | Ileum | 1 |
| Catalase KatE-intracellular protease (EC 1.11.1.6) | Ileum | 2 |
| Glycerol dehydrogenase (EC 1.1.1.6) | Ileum | 1 |
| HP-UF-Rhamnulokinase (EC 2.7.1.5)-DF-L-rhamnose operon regulatory protein RhaS | Ileum | 1 |
| N-acetylmuramoyl-L-alanine amidase (EC 3.5.1.28) | Ileum | 1 |
| Phosphatidate cytidylyltransferase (EC 2.7.7.41) | Ileum | 1 |
| Threonylcarbamoyl-AMP synthase (EC 2.7.7.87) | Ileum | 1 |
| UPF0192 protein YfaS | Ileum | 1 |
| beta-galactosidase (EC 3.2.1.23) | Rectum | 1 |
| Cell division inhibitor Slr1223 (YfcH in EC), contains epimerase/dehydratase and DUF1731 domains | Rectum | 1 |
| Colanic acid biosynthesis glycosyl transferase WcaC | Rectum | 1 |
| Copper/silver efflux RND transporter, transmembrane protein CusA | Rectum | 1 |
| Dienelactone hydrolase family protein | Rectum | 1 |
| DNA gyrase subunit B (EC 5.99.1.3) | Rectum | 1 |
| Glucosamine-6-phosphate deaminase (EC 3.5.99.6) | Rectum | 1 |
| Glutathione S-transferase, omega (EC 2.5.1.18) | Rectum | 1 |
| FIG00638721: HP-no UF or DF | Rectum | 1 |
| Mechanosensitive ion channel | Rectum | 1 |
| Mannonate dehydratase (EC 4.2.1.8) | Rectum | 1 |
| Polyferredoxin NapH (periplasmic nitrate reductase) | Rectum | 1 |
| Possible hydrolase or acyltransferase RutD in novel pyrimidine catabolism pathway | Rectum | 1 |
| Putative alanine/glycine transport protein | Rectum | 1 |
| Taurine ABC transporter, substrate-binding protein TauA | Rectum | 1 |
| Tagatose-1,6-bisphosphate aldolase GatY (EC 4.1.2.40) | Rectum | 1 |

HP, hypothetical protein; UF, upstream feature; DF, downstream feature.

g- ST14

| **Gene associated functions** | **Gut location** | **Frequency** |
| --- | --- | --- |
| LysR-family transcriptional regulator YjiE | Ileum | 1 |
| PTS system, inactive IIB catalytic domain / PTS system, fructose-specific IIB component (EC 2.7.1.202) / PTS system, fructose-specific IIC component | Ileum | 1 |
| HtrA protease/chaperone protein | Rectum | 1 |
| HP-UF-FIG004016: Uncharacterized protein YggN-DF-FIG002060: uncharacterized protein YggL | Rectum | 1 |
| L-2-hydroxyglutarate oxidase (EC 1.1.3.15) | Rectum | 1 |
| Malate synthase G (EC 2.3.3.9) | Rectum | 1 |
| Phosphotriesterase like protein Php | Rectum | 1 |
| Sodium-dependent phosphate transporter | Rectum | 1 |

HP, hypothetical protein; UF, upstream feature; DF, downstream feature.

h- ST91

| **Gene associated functions** | **Gut location** | **Frequency** |
| --- | --- | --- |
| DNA-3-methyladenine glycosylase II (EC 3.2.2.21) | Ileum | 1 |
| Glucose dehydrogenase, PQQ-dependent (EC 1.1.5.2) | Ileum | 1 |
| Glutathione-regulated potassium-efflux system protein KefC | Ileum | 1 |
| Outer membrane lipoprotein component of lipoprotein transport system LolB | Ileum | 1 |
| Trehalose operon transcriptional repressor | Ileum | 1 |
| 5-amino-6-(5-phospho-D-ribitylamino) uracil phosphatase YbjI | Rectum | 1 |
| 23S rRNA (cytidine(2498)-2'-O)-methyltransferase (EC 2.1.1.186) | Rectum | 1 |
| Aconitate hydratase (EC 4.2.1.3) @ 2-methylisocitrate dehydratase (EC 4.2.1.99) | Rectum | 1 |
| Conserved ATP-binding protein YghS | Rectum | 1 |
| Inositol-1-monophosphatase (EC 3.1.3.25) | Rectum | 1 |
| NAD-dependent dihydropyrimidine dehydrogenase subunit PreA (EC 1.3.1.1) | Rectum | 1 |
| tRNA (5-methylaminomethyl-2-thiouridylate)-methyltransferase (EC 2.1.1.61) / FAD-dependent cmnm (5)s(2)U34 oxidoreductase | Rectum | 1 |
| Uncharacterized metal-dependent hydrolase YcfH | Rectum | 1 |
| Uncharacterized protein YdhT | Rectum | 1 |

i- ST491

| **Gene associated functions** | **Gut location** | **Frequency** |
| --- | --- | --- |
| 3'-to-5' exoribonuclease RNase R | Ileum | 1 |
| Acyl-coenzyme A dehydrogenase FadE (EC 1.3.8.-) | Ileum | 1 |
| Exodeoxyribonuclease I (EC 3.1.11.1) | Ileum | 1 |
| Murein DD-endopeptidase MepH | Ileum | 1 |
| Predicted ATP-dependent endonuclease of the OLD family, YbjD subgroup | Ileum | 1 |
| Putative capsular polysaccharide transport protein YegH | Ileum | 1 |
| Ribulokinase (EC 2.7.1.16) | Ileum | 1 |
| Trehalose-6-phosphate hydrolase (EC 3.2.1.93) | Ileum | 1 |
| 23S rRNA (uracil (1939)-C (5))-methyltransferase (EC 2.1.1.190) | Rectum | 1 |
| Putative transport protein | Rectum | 1 |
| Signal recognition particle receptor FtsY | Rectum | 1 |
| UPF0060 membrane protein YnfA | Rectum | 1 |

j- ST550

| **Gene associated functions** | **Gut location** | **Frequency** |
| --- | --- | --- |
| Aspartate ammonia-lyase (EC 4.3.1.1) | Ileum | 1 |
| [Citrate [pro-3S]-lyase] ligase (EC 6.2.1.22) | Rectum | 1 |
| Alkylation response protein AidB, acyl-CoA dehydrogenase family | Rectum | 1 |
| Antigen 43 | Rectum | 1 |
| Serine palmitoyl transferase (EC 2.3.1.50) | Rectum | 1 |

k- ST636

| **Gene associated functions** | **Gut location** | **Frequency** |
| --- | --- | --- |
| DNA primase DnaG | Ileum | 1 |
| Gamma-glutamyl transpeptidase (EC 2.3.2.2) @ Glutathione hydrolase (EC 3.4.19.13) | Ileum | 1 |
| HP-UF-Membrane protein FdrA-DF-Isochorismatase (EC 3.3.2.1) | Ileum | 1 |
| Inner membrane protein YcfT | Ileum | 1 |
| Inner membrane protein YfdC | Ileum | 1 |
| Rhamnulokinase (EC 2.7.1.5) | Ileum | 1 |
| Septum-associated cell division protein DamX | Ileum | 1 |
| Aspartate--ammonia ligase (EC 6.3.1.1) | Rectum | 1 |
| Methyl-accepting chemotaxis protein I (serine chemoreceptor protein) | Rectum | 1 |
| Phenylalanyl-tRNA synthetase beta chain (EC 6.1.1.20) | Rectum | 1 |
| Putative acid--amine ligase YjfC | Rectum | 1 |
| Uncharacterized protein YeeL | Rectum | 1 |

HP, hypothetical protein; UF, upstream feature; DF, downstream feature.

l- ST1193

| **Gene associated functions** | **Gut location** | **Frequency** |
| --- | --- | --- |
| 2-ketoaldonate reductase, broad specificity (EC 1.1.1.215) | Ileum | 1 |
| Histidinol dehydrogenase (EC 1.1.1.23) | Ileum | 1 |
| L-tartrate/succinate antiporter | Ileum | 1 |
| Trehalase (EC 3.2.1.28) @ Periplasmic trehalase (EC 3.2.1.28) | Ileum | 1 |
| HP-UF-D-galactarate transporter @ D-glucarate transporter-DF-D-galactarate dehydratase (EC 4.2.1.42) | Rectum | 1 |

HP, hypothetical protein; UF, upstream feature; DF, downstream feature.

m- ST11174

| **Gene associated functions** | **Gut location** | **Frequency** |
| --- | --- | --- |
| Colanic acid biosynthesis protein WcaM | Ileum | 1 |
| Polysaccharide pyruvyl transferase | Ileum | 1 |
| Hexuronate utilization operon transcriptional repressor ExuR | Rectum | 1 |

**Table S2.** Components of Biolog plates and phenotypic performance by *E. coli* (based on OD value representing metabolic performance of *E. coli*).

| **Biolog GenIII substrates** | | | |
| --- | --- | --- | --- |
| A2) Dextrin | A3) D-Maltose | A4) D-Trehalose | A5) D-Cellobiose |
| A6) Gentiobiose | A7) Sucrose | A8) D-Turanose | A9) Stachyose |
| A11) pH 6 | A12) pH 5 | B1) D-Raffinose | B2) α-D-Lactose |
| B3) D-Melibiose | B4) β-Methyl-D-Glucoside | B5) D-Salicin | B6) N-Acetyl-D-Glucosamine |
| B7) N-Acetyl-β-D-Mannosamine | B8) N-Acetyl-D-Galactosamine | B9) N-Acetyl Neuraminic Acid | B10) 1% NaCl |
| B11) 4% NaCl | B12) 8% NaCl | C1) α-D-Glucose | C2) D-Mannose |
| C3) D-Fructose | C4) D-Galactose | C5) 3-Methyl Glucose | C6) D-Fucose |
| C7) L-Fucose | C8) L-Rhamnose | C9) Inosine | C10) 1% Sodium Lactate |
| C11) Fusidic Acid | C12) D-Serine | D1) D-Sorbitol | D2) D-Mannitol |
| D3) D-Arabitol | D4) myo-Inositol | D5) Glycerol | D6) D-Glucose-6-PO4 |
| D7) D-Fructose-6-PO_4_ | D8) D-Aspartic Acid | D9) D-Serine | D10) Troleandomycin |
| D11) Rifamycin SV | D12) Minocycline | E1) Gelatin | E2) Glycyl-L-Proline |
| E3) L-Alanine | E4) L-Arginine | E5) L-Aspartic Acid | E6) L-Glutamic Acid |
| E7) L-Histidine | E8) L-Pyroglutamic Acid | E9) L-Serine | E10) Lincomycin |
| E11) Guanidine HCl | E12) Niraproof 4 | F1) Pectin | F2) D-Galacturonic Acid |
| F3) L-Galactonic Acid Lactone | F4) D-Gluconic Acid | F5) D-Glucuronic Acid | F6) Glucuronamide |
| F7) Mucic Acid | F8) Quinic Acid | F9) D-Saccharic Acid | F10) Vancomycin |
| F11) Tetrazolium Violet | F12) Tetrazolium Blue | G1) p-Hydroxy-Phenylacetic Acid | G2) Methyl Pyruvate |
| G3) D-Lactic Acid Methyl Ester | G4) L-Lactic Acid | G5) Citric Acid | G6) α-Keto-Glutaric Acid |
| G7) D-Malic Acid | G8) L-Malic Acid | G9) Bromo-Succinic Acid | G10) Nalidixic Acid |
| G11) Lithium Chloride | G12) Potassium Tellurite | H1) Tween 40 | H2) γ-Amino-Butyric Acid |
| H3) α-Hydroxy-Butyric Acid | H4) β-Hydroxy-D, L-Butyric Acid | H5) α-Keto-Butyric Acid | H6) Acetoacetic Acid |
| H7) Propionic Acid | H8) Acetic Acid | H9) Formic Acid | H10) Aztreonam |
| H11) Sodium Butyrate | H12) Sodium Bromate |  |  |

OD values greater than negative controls were considered for inclusion in data analysis. This restriction resulted in elimination of 16 carbon source and one chemical reactions from the analysis (red color). Also, there were 32 carbon source and 15 chemicals readings where both the terminal ileum and rectum strains grew but did not vary from each other and were ultimately excluded from the analysis (blue color). If the terminal ileum and rectum strains grew and varied significantly were considered for analysis (black color). Letter indicates the well number in MicroPlate.

**
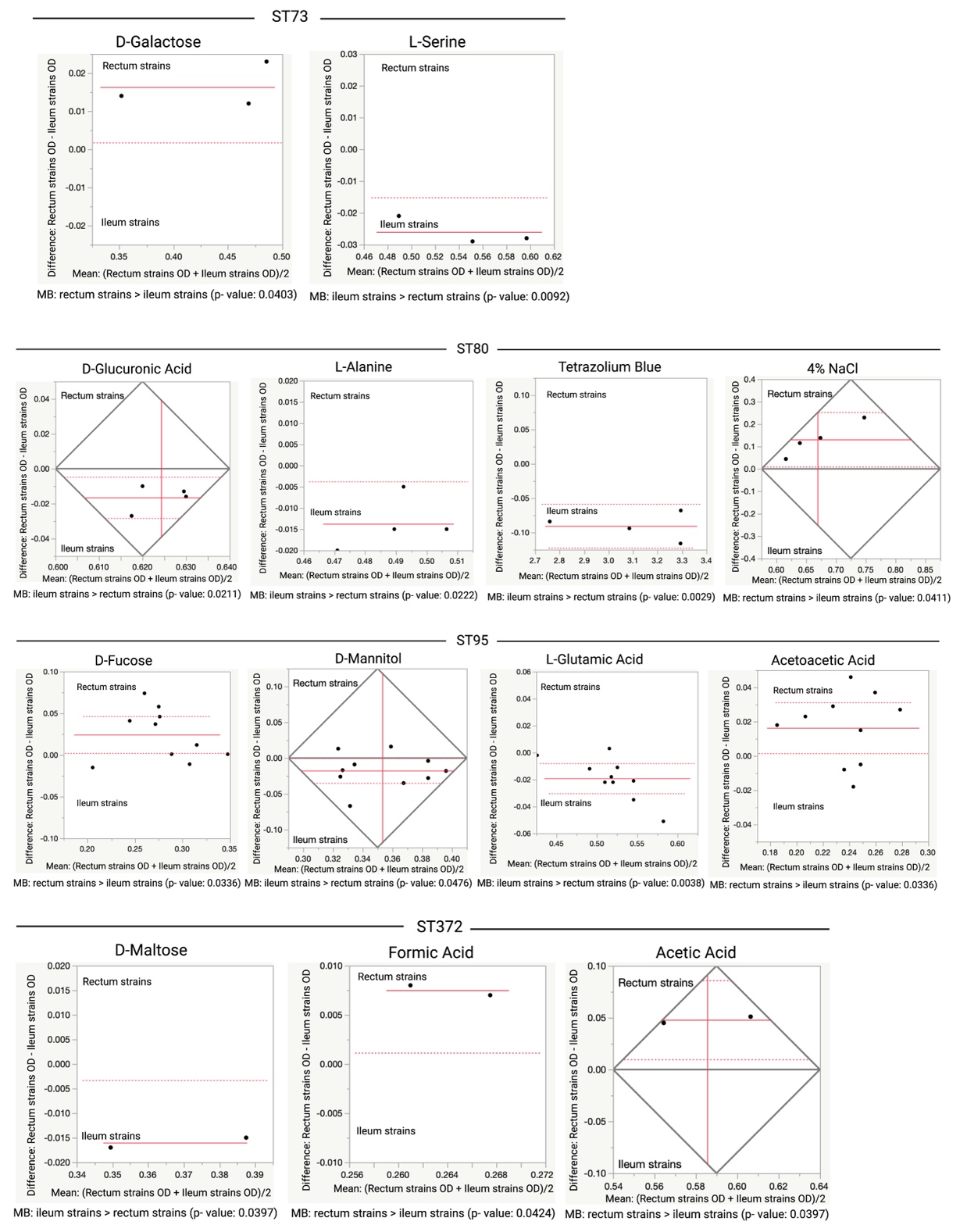
**

**Figure S4.** Comparison of OD values of the terminal ileum (Ti) and rectum (R) strains of *E. coli* ST73, ST80, ST95 and ST372 grown in Biolog carbon sources and chemicals. Data shows only those carbon sources and chemicals where the terminal ileum and rectum strains showed significant variation. Paired t-test was used for this analysis. D, difference; M, mean; MD, mean difference; G, growth. Each dot indicates mean (x-axis) and difference (y-axis) of each clone-pair. The red line or the intersected point of red lines indicates the mean difference (y-axis) which fall towards either the rectum or ileum strains and the red dashed lines indicates upper and lower bounds (at 95% confidence interval). ‘>’, indicates which type of strains show the higher growth.


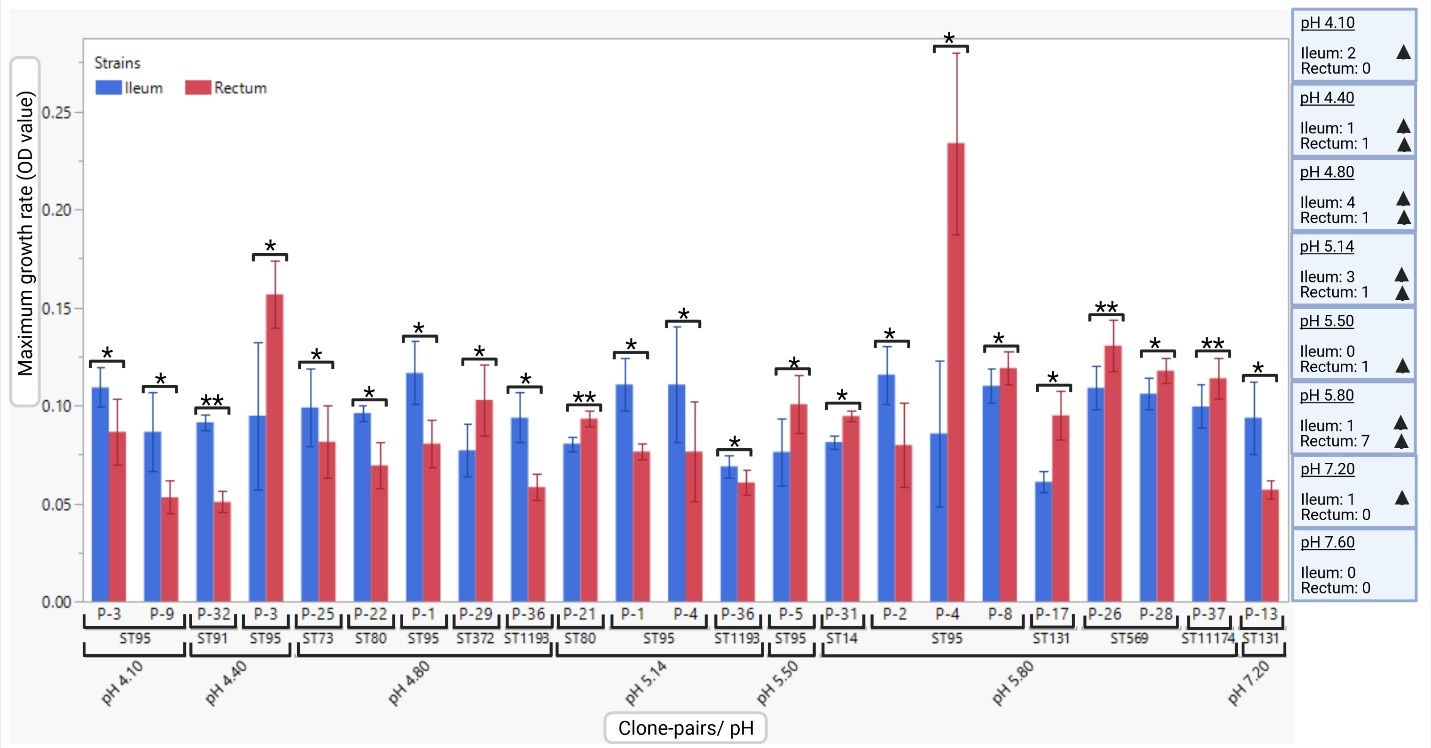


Figure S5. Clone pair analysis for maximum growth rate comparison between the terminal ileum and rectum strains of *E. coli* grown in different pHs. *, p ≤0.05; ** p ≤0.01. The triangle arrow-up icon in the right-hand side boxes represents the number(s) of clone pairs at each pH that showed significantly elevated maximum growth rate either in the terminal ileum or rectum strains.


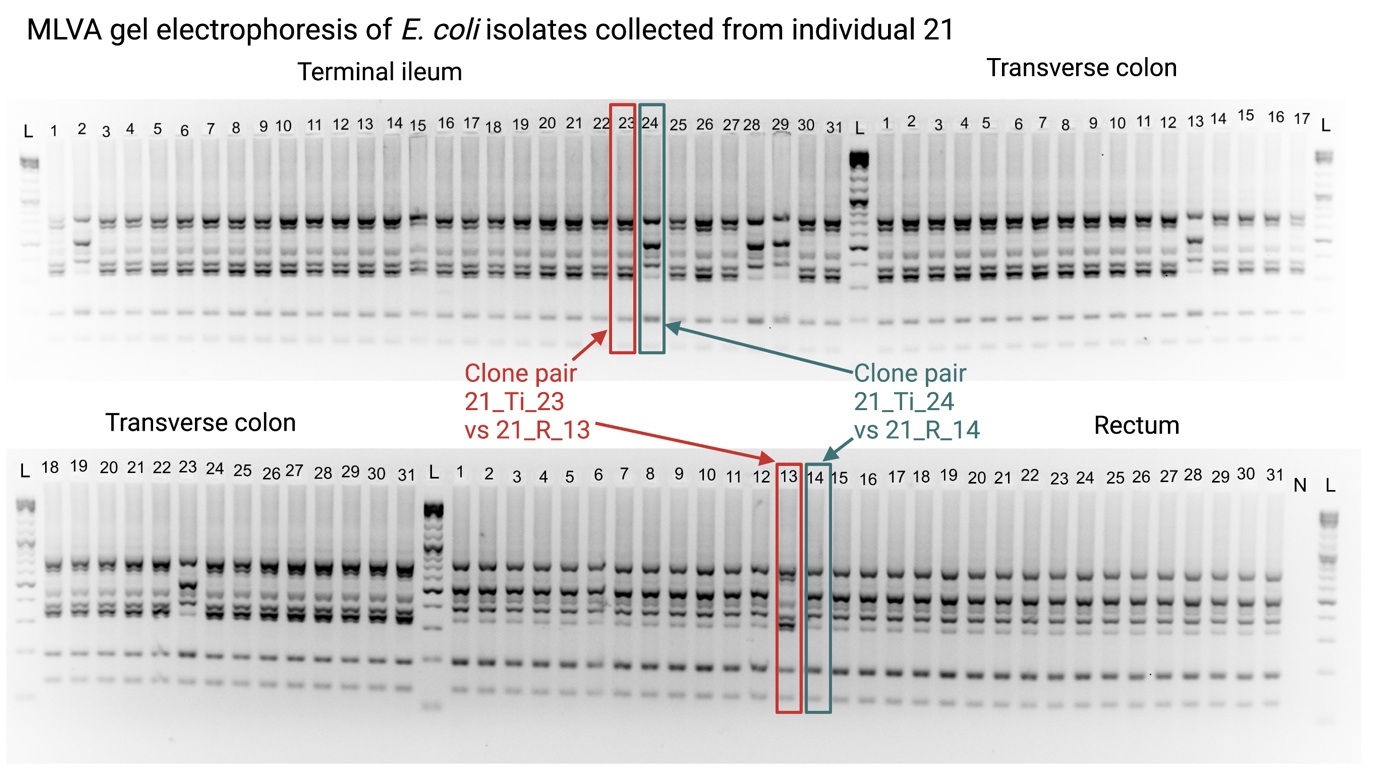


**Figure S6.** An example of selecting clone pairs of *E. coli* from individual 21. MLVA gel electrophoresis shows, there are two strains in each of the gut locations (terminal ileum, transverse colon, and rectum). The strains (indicated by red colour) numbered 23 and 13 of terminal ileum and rectum respectively, gave similar gel band and belonged to phylogroup B2, and they were referred as a clone pair. Also, the strains (indicated by green colour) numbered 24 and 14 of terminal ileum and rectum respectively, gave similar gel band and belonged to phylogroup B2, and were referred as another clone pair. In total, analysing 93 isolates from terminal ileum, transverse colon, and rectum of individual No. 21 gave two different clone pairs. Each number of gel band indicates the colony number of the isolate. A list of clone pairs isolated from total individuals with their demographics is provided in Table S3.

**Table S3.** *E. coli* clone pairs and individuals’ demographic information.

| **SL no** | **Individual** | **Pair** | **Isolate ID** | **Location of isolate collection** | **Age (years)** | **Sex** | **Disease status** |
| --- | --- | --- | --- | --- | --- | --- | --- |
| 1 | 5 | P-1 | 5_Ti_1 | Ileum | 61 | F | N |
|  |  |  | 5_R_3 | Rectum |  |  |  |
| 2 | 7 | P-11 | 7_Ti_10 | Ileum | 70 | F | N |
|  |  |  | 7_R_5 | Rectum |  |  |  |
| 3 | 8 | P-23 | 8_Ti_11 | Ileum | 59 | F | N |
|  |  |  | 8_R_11 | Rectum |  |  |  |
| 4 | 9 | P-12 | 9_Ti_6 | Ileum | 67 | M | N |
|  |  |  | 9_R_4 | Rectum |  |  |  |
|  |  | P-2 | 9_Ti_7 | Ileum |  |  |  |
|  |  |  | 9_R_11 | Rectum |  |  |  |
| 5 | 11 | P-24 | 11_Ti_11 | Ileum | 52 | M | N |
|  |  |  | 11_R_4 | Rectum |  |  |  |
| 6 | 12 | P-13 | 12_Ti_16 | Ileum | 64 | M | N |
|  |  |  | 12_R_4 | Rectum |  |  |  |
| 7 | 13 | P-14 | 13_Ti_12 | Ileum | 68 | M | N |
|  |  |  | 13_R_4 | Rectum |  |  |  |
| 8 | 14 | P-32 | 14_Ti_10 | Ileum | 51 | F | N |
|  |  |  | 14_R_6 | Rectum |  |  |  |
| 9 | 16 | P-37 | 16_Ti_1 | Ileum | 36 | F | UC |
|  |  |  | 16_R_2 | Rectum |  |  |  |
| 10 | 17 | P-3 | 17_Ti_1 | Ileum | 33 | M | N |
|  |  |  | 17_R_2 | Rectum |  |  |  |
| 11 | 19 | P-35 | 19_Ti_1 | Ileum | 43 | M | N |
|  |  |  | 19_R_1 | Rectum |  |  |  |
| 12 | 21 | P-4 | 21_Ti_23 | Ileum | 35 | F | CD |
|  |  |  | 21_R_13 | Rectum |  |  |  |
|  |  | P-25 | 21_Ti_24 | Ileum |  |  |  |
|  |  |  | 21_R_14 | Rectum |  |  |  |
| 13 | 23 | P-15 | 23_Ti_15 | Ileum | 54 | M | N |
|  |  |  | 23_R_21 | Rectum |  |  |  |
| 14 | 24 | P-33 | 24_Ti_1 | Ileum | 54 | M | N |
|  |  |  | 24_R_1 | Rectum |  |  |  |
| 15 | 25 | P-5 | 25_Ti_27 | Ileum | 62 | F | N |
|  |  |  | 25_R_1 | Rectum |  |  |  |
| 16 | 27 | P-29 | 27_Ti_2 | Ileum | 28 | F | CD |
|  |  |  | 27_R_4 | Rectum |  |  |  |
| 17 | 29 | P-16 | 29_Ti_13 | Ileum | 28 | F | N |
|  |  |  | 29_R_3 | Rectum |  |  |  |
| 18 | 30 | P-6 | 30_Ti_4 | Ileum | 38 | F | N |
|  |  |  | 30_R_4 | Rectum |  |  |  |
| 19 | 33 | P-19 | 33_Ti_4 | Ileum | 57 | M | CD |
|  |  |  | 33_R_4 | Rectum |  |  |  |
| 20 | 35 | P-7 | 35_Ti_26 | Ileum | 78 | M | N |
|  |  |  | 35_R_15 | Rectum |  |  |  |
| 21 | 36 | P-36 | 36_Ti_2 | Ileum | 71 | M | N |
|  |  |  | 36_R_5 | Rectum |  |  |  |
| 22 | 42 | P-17 | 42_Ti_1 | Ileum | 39 | M | N |
|  |  |  | 42_R_1 | Rectum |  |  |  |
|  |  | P-10 | 42_Ti_3 | Ileum |  |  |  |
|  |  |  | 42_R_25 | Rectum |  |  |  |
| 23 | 44 | P-20 | 44_Ti_1 | Ileum | 55 | M | N |
|  |  |  | 44_R_1 | Rectum |  |  |  |
| 24 | 45 | P-30 | 45_Ti_1 | Ileum | 65 | F | N |
|  |  |  | 45_R_17 | Rectum |  |  |  |
| 25 | 47 | P-21 | 47_Ti_4 | Ileum | 57 | F | N |
|  |  |  | 47_R_3 | Rectum |  |  |  |
| 26 | 48 | P-26 | 48_Ti_2 | Ileum | 56 | M | N |
|  |  |  | 48_R_2 | Rectum |  |  |  |
| 27 | 50 | P-27 | 50_Ti_3 | Ileum | 27 | F | N |
|  |  |  | 50_R_1 | Rectum |  |  |  |
| 28 | 19# | P-31 | 73: 19_2_Ti6 | Ileum | 26 | F | CD |
|  |  |  | 78: 19_1_R4 | Rectum |  |  |  |
| 29 | 33# | P-18 | 137: 33_1_Ti5 | Ileum | 53 | M | CD |
|  |  |  | 144: 33_1_R5 | Rectum |  |  |  |
| 30 | 39# | P-8 | 154: 39_2_Ti18 | Ileum | 28 | F | CD |
|  |  |  | 157: 39_1_R4 | Rectum |  |  |  |
| 31 | 46# | P-9 | 177: 46_1_Ti2 | Ileum | 30 | M | UC |
|  |  |  | 182: 46_1_R5 | Rectum |  |  |  |
| 32 | 52# | P-34 | 212: 52_2_Ti10 | Ileum | 29 | F | N |
|  |  |  | 219: 52_2_R1 | Rectum |  |  |  |
| 33 | 60# | P-22 | 266: 60_1_Ti1 | Ileum | 32 | F | UC |
|  |  |  | 269: 60_2_R4 | Rectum |  |  |  |
| 34 | 70# | P-28 | 347: 70_2_Ti12 | Ileum | 21 | M | CD |
|  |  |  | 356: 70_2_R11 | Rectum |  |  |  |

F, Female; M, Male; N, Normal; CD, Crohn’s disease; UC, Ulcerative colitis; #, *E. coli* collection from Gordon *et al*. All strains were re-confirmed belonging to B2 phylogroup via ClermonTyping.

**Table S4.** B2 reference strain selected for each ST with their accession number.

| **ST** | **Reference strains’ accession number** |
| --- | --- |
| ST95, ST2619 | NC_011742, NC_011747 |
| ST73 | NZ_CP016497, NZ_CP016498 |
| ST131, ST91 | NZ_HG941718, NZ_HG941719, NZ_HG941720 |
| ST569 | NZ_CP023388, NZ_CP023389 |
| ST372, ST80, ST11174, ST550, ST14, ST1193, ST636 | CP012379.1 |
| ST491 | NC_011993 |
